# Supplementary material for: Learning to represent signals spike by spike
Source: PLoS Comput Biol. 2020 Mar 16;16(3):e1007692. doi: 10.1371/journal.pcbi.1007692 (PMC7135338; doi:10.1371/journal.pcbi.1007692)
Supplement: S1 Text — While it is referenced section by section, it can also be read as a separate, more technical explanation of the learning rules. (PDF) [file pcbi.1007692.s001.pdf]

Supplementary Material

Learning to represent signals spike by spike

Wieland Brendel, Ralph Bourdoukan, Pietro Vertechi,
Christian K. Machens and Sophie Denève

Contents

1 The efficient coding objective **4**
1.1 Inputs and outputs 4
1.2 The linear readout 4
1.3 Efficient coding 5
1.4 The cost terms 6

2 Networks of integrate-and-fire neurons **7**

3 Networks that obey 'error-driven coding' **8**

4 Networks optimized for a fixed decoder **9**

5 The optimal decoder **11**
5.1 Solution for arbitrary networks 11
5.2 Solution under length constraints 11
5.3 The optimal decoder for the optimal network architecture 12

6 The four conditions of learning **13**
6.1 Condition 1: Spike-by-spike balance 13
6.2 Condition 2: Recurrent weights need to be $\mathbf{\Omega} = -\mathbf{FD}$ 13
6.3 Interlude: the importance of quadratic costs 13
6.4 Condition 3: Feedforward weights need to mimic $\mathbf{D}$ 14
6.5 Condition 4: Decoder needs to minimize the loss 14
6.6 A plan of action for learning all the weights 14

7 Recurrent weights: current-based learning **15**
7.1 Condition 1: Recurrent weights learn to balance 15
7.2 Condition 2: Spike-by-spike balance yields error-driven coding . . 16

8 Feedforward weights: current-based learning **18**
8.1 Quadratic costs modify recurrent learning 18
8.2 Condition 3: Feedforward weights learn to mimic the decoder . . 18
8.3 Condition 4: Learning rules minimize loss function 19
8.4 Conclusions and biological realism reconsidered 20

9 Recurrent weights: Voltage-based learning **21**

9.1 Condition 1: Recurrent weights learn to minimize voltage fluctuations . . . 21
9.2 Condition 2: Recurrent weights reach error-driven-coding architecture . . . 22
9.3 Condition 2: Convergence proof . . . 24
9.4 Learning with L2 costs . . . 25

10 Feedforward weights: Voltage-based learning . . . 26
10.1 Condition 3: Feedforward weights mimic decoder . . . 26
10.2 Condition 4: Feedforward weights reach optimum . . . 27

11 Learning with L1 costs . . . 28
11.1 Scaling of the synaptic weights . . . 28
11.2 Modified learning of recurrent weights . . . 28
11.3 Modified learning for feedforward weights . . . 29
11.4 Simplifying assumptions and final learning rules . . . 29

12 Learning rules for non-whitened inputs . . . 31

13 Learning in the EI network . . . 33
13.1 Voltage dynamics . . . 33
13.2 Learning the synapses . . . 33

14 Numerical Simulations . . . 35
14.1 Network Dynamics . . . 35
14.2 Learning . . . 35
14.3 Initialization . . . 38
14.4 Tuning Curves . . . 38
14.5 Fano Factor and Coefficient of Variation . . . 40
14.6 Simulation of speech signal learning . . . 40

Variable	Description
$\mathbf{c}$	M-dimensional vector of input signals
c_j	j-th input signal
$\mathbf{D}$	decoder matrix
$\mathbf{D}_k$	k-th column of $\mathbf{D}$, or decoding vector of the k-th neuron
D_{jk}	element of the $\mathbf{D}$; or contribution of the k-th neuron to the j-th signal
$\mathbf{F}$	matrix of feedforward weights
$\mathbf{F}_i$	i-th row of $\mathbf{F}$, or vector of feedforward weights of the i-th neuron
F_{ij}	element of $\mathbf{F}$, or feedforward weight of j-th signal onto i-th neuron
M	number of input signals
N or N_E	number of excitatory neurons
N_I	number of inhibitory neurons
$\mathbf{o}$	N-dimensional vector of output spike trains
o_i	output spike train of the i-th neuron
$\mathbf{r}$	N-dimensional vector of filtered output spike trains
r_i	filtered output spike train of the i-th neuron
$\mathbf{T}$	N-dimensional vector of voltage thresholds
T_i	voltage threshold of the i-th neuron
$\mathbf{V}$	N-dimensional vector of voltages
V_i	voltage of the i-th neuron
λ	leak parameter for voltages and filtering of spike trains
μ	parameter for quadratic (L2) cost term
ν	parameter for linear (L1) cost term
$\mathbf{\Omega}$	matrix of recurrent weights
$\mathbf{\Omega}_i$	i-th row of $\mathbf{\Omega}$, or vector of recurrent weights of the i-th neuron
Ω_{ik}	element of $\mathbf{\Omega}$, or recurrent weight of k-th neuron onto i-th neuron

Table 1: Mathematical notation.

1 The efficient coding objective

In this section, we will first define the inputs and outputs of the network in more detail, develop the exact nature of the linear readout, and then specify the efficient coding objective, which is the core objective the network will seek to minimize.

1.1 Inputs and outputs

We consider a recurrent neural network with N_E excitatory and N_I inhibitory neurons (compare Figure 1Ai in the main paper). The network receives a set of time-varying inputs $\mathbf{c}(t) = \big(c_1(t), c_2(t), \ldots, c_M(t)\big)$ and produces a set of spike train outputs from the excitatory population, $\mathbf{o}(t) = \big(o_1(t), o_2(t), \ldots, o_{N_E}(t)\big)$. Each spike train is as a sum of Dirac delta functions, $o(t) = \sum_{t_k} \delta(t - t_k)$, where t_k are the spike times.

We furthermore define filtered versions of the input and output signals. First, the filtered input signal, $\mathbf{x}(t)$, is given by

$$\dot{\mathbf{x}}(t) = -\lambda \mathbf{x}(t) + \mathbf{c}(t), \tag{S.1}$$

and the filtered spike trains of the excitatory neurons are given by

$$\dot{\mathbf{r}}(t) = -\lambda \mathbf{r}(t) + \mathbf{o}(t). \tag{S.2}$$

Here, the parameter λ sets the decay rate of the respective variables. We note that for slowly changing signals, $\mathbf{x}(t)$ and $\mathbf{c}(t)$ are just scaled versions of each other. This is the scenario most applicable to our work, and we therefore refer to both variables as 'input signals'. Indeed, in the main text we did not distinguish between $\mathbf{c}(t)$ and $\mathbf{x}(t)$, but will do so here to be mathematically exact. We assume that these input signals are distributed according to some distribution $q(\mathbf{x})$. We will assume that this distribution is 'white', i.e., that its covariance matrix is the identity, an assumption we will relax in Section S10.

Each filtered spike train can be viewed as a sum over postsynaptic potentials. We will sometimes refer to these filtered spike trains as 'instantaneous firing rates' or simply 'firing rates'. Note that the variables $r_i(t)$ have units of firing rates scaled by a factor $1/\lambda$, a definition that slightly deviates from [1]. The spike trains of the inhibitory interneurons are not considered to be part of the output, and will be ignored for now.

1.2 The linear readout

We will assume that a downstream area will seek to construct an estimate $\hat{\mathbf{x}}(t)$ of the input signal from a simple weighted sum of the filtered output spike trains,[1]

$$\begin{aligned} \hat{\mathbf{x}} &= \mathbf{D}\mathbf{r} \qquad \text{(S.3)} \\ &= \sum_{n=1}^{N_E} \mathbf{D}_n r_n \end{aligned}$$

[1] Please note that we will generally drop the explicit notion of time-dependence to streamline the presentation, and so we here write $\hat{\mathbf{x}}$ instead of $\hat{\mathbf{x}}(t)$ and $\mathbf{r}$ instead of $\mathbf{r}(t)$.

where $\mathbf{D}$ is an $M \times N_E$ matrix of decoder weights, and $\mathbf{D}_n$ are the columns of this matrix. The vector $\mathbf{D}_n$ summarizes the contribution of neuron n to the reconstruction of the signal.

We make two observations: First, the equation for the linear readout is simply the vectorized version of Equation (2) in the main paper. Second, we can use (S.2) to obtain a differential equation for this readout,

$$\dot{\hat{\mathbf{x}}} = -\lambda \hat{\mathbf{x}} + \mathbf{D}\mathbf{o},$$

which will become useful further below.

1.3 Efficient coding

We can measure the quality of any readout by averaging its performance over a time interval T. To denote time averages of a quantity $z(t)$, we will use angular brackets so that $\langle z(t) \rangle_t = \frac{1}{T}\int_0^T dt\, z(t)$. With this in mind, we define the following loss function,

$$\begin{aligned} L &= \langle \ell(t) \rangle_t \\ &\equiv \left\langle \|\mathbf{x}(t) - \hat{\mathbf{x}}(t)\|^2 + C\big(\mathbf{r}(t)\big) \right\rangle_t , \end{aligned}$$

which is simply the vectorized version of Equation (3) in the main paper. Here the first term inside the brackets is a quadratic measure of the reconstruction error, the second term is a cost term on the firing rates.

If the time interval T over which this loss is averaged is large compared to the time-scale of the input, then the distribution of inputs $\mathbf{c}$ during this interval is approximately the same as the true input distribution $q(\mathbf{c})$. Hence, for large T we essentially sample the loss evenly over the distribution $q(\mathbf{c})$ of all possible inputs $\mathbf{c}$ (and hence over the distribution of input signals $q(\mathbf{x})$, since the signals $\mathbf{x}$ are filtered versions of $\mathbf{c}$). To denote expectation values of a quantity $z(\mathbf{x})$ with respect to the distribution $q(\mathbf{x})$, we will again use angular brackets, writing $\langle z(\mathbf{x}) \rangle_{q(\mathbf{x})} = \int d\mathbf{x} q(\mathbf{x}) z(\mathbf{x})$. Accordingly, we can rewrite (S.4) as an estimate of the expected loss over the inputs,[2]

$$\begin{aligned} L &= \langle \ell(\mathbf{x}) \rangle_{q(\mathbf{x})} \\ &= \left\langle \|\mathbf{x} - \hat{\mathbf{x}}\|^2 + C(\mathbf{r}) \right\rangle_{q(\mathbf{x})} . \end{aligned} \tag{S.4}$$

For ease of notation we will typically suppress the difference between these two formulations and simply use angular brackets, $\langle z \rangle$, to denote averaging of the variable z over either time or input signals. Similarly, we will write

$$\ell = \|\mathbf{x} - \hat{\mathbf{x}}\|^2 + C(\mathbf{r}) \tag{S.5}$$

to refer either to the time-dependent loss $\ell(t)$ or the signal-dependent loss $\ell(\mathbf{x})$.

[2]We here assume that the distribution $q(\mathbf{x})$ is white, i.e., its covariance matrix is the identity. For non-white signals, it is advantageous to modify the definition of the loss, and we will discuss this more general case in section 12.

1.4 The cost terms

The cost term, $C(\mathbf{r})$, allows us to assign a 'cost' to the representation (in terms of filtered spike trains) chosen by a particular network. Typical choices for the cost-term are $C(\mathbf{r}) = \sum_i r_i^2 = \|\mathbf{r}\|^2$ or $C(\mathbf{r}) = \sum_i |r_i| = \|\mathbf{r}\|_1$. For concreteness, we adopt a linear sum of the two, but many results and intuitions directly generalize to other cost functions. The objective (S.5) then reads

$$\ell = \|\mathbf{x} - \hat{\mathbf{x}}\|^2 + \mu \|\mathbf{r}\|^2 + \nu \|\mathbf{r}\|_1 . \qquad \text{(S.6)}$$

To understand the influence of the cost terms, we imagine that there are (many) more neurons than independent inputs, i.e., $N > M$. In the absence of costs, and assuming real-valued firing rates $\mathbf{r}$ and constant inputs $\mathbf{x}$ for a moment, we see that many possible combinations of firing rates can minimize the objective, (S.4). Possible solutions are those in which only a few neurons fire with high rates (sparse regime), and those in which many neurons fire with fairly low firing rates (dense regime).

The cost term allows us to define which firing rate patterns have a higher efficiency. The typical choice to enforce sparse population responses is a so-called *L1-cost*, $C(\mathbf{r}) = \|\mathbf{r}\|_1 = \sum_n |r_n|$, and the typical choice to enforce a dense population code is a so-called *L2-cost* $C(\mathbf{r}) = \|\mathbf{r}\|_2^2 = \sum_n r_n^2$. Many other costs such as slowness, group sparsity and others have been extensively discussed in the literature, especially in the context of regularization. We here adopt a linear sum of L1- and L2-cost for the rest of this manuscript, but the generalization to other cost functions is possible with typically few modifications (see also [1] for a more detailed discussion).

We note that algorithms such as principal component analysis (PCA) and independent component analysis (ICA [17]) can be formulated as special cases of this general class of learning problems. In particular, [21] relates ICA with efficient coding in the presence of a sparsity cost.

2 Networks of integrate-and-fire neurons

In this section, we will briefly describe the network equations and introduce their vectorized and integrated versions, which will become important in later sections.

The dynamics of the membrane voltages is given by Equation (1) in the main paper. We will rewrite this equation in a slightly more general form by introducing two modifications. First, we introduce a leak parameter λ which determines the strength of the voltage leak term, and which is identical to the parameter used in the definition of the filtered spike trains, (S.2). We furthermore use the more general input term $\mathbf{c}(t) = \dot{\mathbf{x}}(t) + \lambda\mathbf{x}(t)$, as explained in Section S1. For slowly changing inputs, $\mathbf{c}(t)$ reduces to $\lambda\mathbf{x}(t)$. Using a more compact vector-notation for the synaptic weights, we obtain

$$\dot{V}_n(t) = -\lambda V_n(t) + \mathbf{F}_n^\top \mathbf{c}(t) + \mathbf{\Omega}_n^\top \mathbf{o}(t).$$

Here the neuron index n runs from $1 \dots N$. The vector of feedforward weights, $\mathbf{F}_n$, is M-dimensional just as the input signal, and the vector of recurrent weights, $\mathbf{\Omega}_n$, is N-dimensional just as the network size. (We note that the recurrent weights can be both excitatory or inhibitory. The treatment of the full EI network can be found in section 13.)

We can further compactify the notation by combining the synaptic weights of the individual neurons into the connectivity matrices of the whole network. We define the $N \times M$ matrix of feedforward weights as $\mathbf{F} = [\mathbf{F}_1, \mathbf{F}_2, \dots, \mathbf{F}_N]^\top$ and the $N \times N$ matrix of recurrent weights as $\mathbf{\Omega} = [\mathbf{\Omega}_1, \dots, \mathbf{\Omega}_N]^\top$.[3] With this notation, we can write the network equations as

$$\dot{\mathbf{V}} = -\lambda\mathbf{V} + \mathbf{F}\mathbf{c} + \mathbf{\Omega}\mathbf{o}, \tag{S.7}$$

where we left out the references to time for brevity.

Finally, we can formally integrate the differential equation, using (S.1) and (S.2), to obtain

$$V_n = \mathbf{F}_n^\top \mathbf{x} + \mathbf{\Omega}_n^\top \mathbf{r}. \tag{S.8}$$

or

$$\mathbf{V} = \mathbf{F}\mathbf{x} + \mathbf{\Omega}\mathbf{r}. \tag{S.9}$$

Note that this integration does not constitute an explicit solution to the differential equation, since the instantaneous firing rates, $\mathbf{r}(t)$, appear on the r.h.s. However, the integration highlights the particular relation between the voltages and the filtered spike trains, which will become useful further below.

[3] Please note that $\mathbf{F}_n$ and $\mathbf{\Omega}_n$ become *rows* of the respective connectivity matrices. In contrast, the decoding vector $\mathbf{D}_n$ denotes a *column* of the decoder matrix $\mathbf{D}$. Nonetheless, all vectors, including these two, are assumed to be column vectors.

3 Networks that obey 'error-driven coding'

In this section, we will briefly describe networks whose recurrent connectivity is given by $\mathbf{\Omega} = -\mathbf{F}\mathbf{D}$, where $\mathbf{F}$ is the feedforward weight matrix, and $\mathbf{D}$ is an (a priori) unknown decoder matrix. These considerations will lead us to two conditions for the learning of the recurrent weights.

To motivate this architecture, we first investigate the scenario in which the input signal can be properly reconstructed from the network output with some decoder $\mathbf{D}$. We will simply assume that the network connectivity is in *some* state in which *some* decoder $\mathbf{D}$ will properly do the job, in which case the reconstruction error should be very small, so that $\mathbf{x} - \hat{\mathbf{x}} \approx 0$.

From the point of view of a single neuron, this reconstruction error is inaccessible. Indeed, the n-th neuron only receives a small part of the input signal, namely the input signal as seen through the lense of its feedforward weights, $\mathbf{F}_n^\top \mathbf{x}$. However, the reconstruction error ε_n for this part of the input signal should, of course, likewise be close to zero so that

$$\begin{aligned}\varepsilon_n &= \mathbf{F}_n^\top \mathbf{x} - \mathbf{F}_n^\top \hat{\mathbf{x}} \\ &= \mathbf{F}_n^\top \mathbf{x} - \mathbf{F}_n^\top \mathbf{D}\mathbf{r} \\ &\approx 0.\end{aligned}$$

Our key insight is now that this latter equation will be identical to the voltage equation, (S.8), if we assume that $\varepsilon_n = V_n \approx 0$ and $\mathbf{\Omega}_n^\top = -\mathbf{F}_n^\top \mathbf{D}$. We note that these are two *necessary* conditions for the network to properly represent the input signals. In matrix notation, we can summarize these two conditions as

Condition 1: $V_n \approx 0$ for all n

Condition 2: $\mathbf{\Omega} = -\mathbf{F}\mathbf{D}$

4 Networks optimized for a fixed decoder

As explained in the last two sections, the connectivity $\mathbf{\Omega} = -\mathbf{FD}$ leads to the voltage equation $\mathbf{V} = \mathbf{Fx} - \mathbf{FDr}$. To gain a better understanding of this voltage equation, and gain a condition for the learning of the feedforward weights, we will here recap the results of [1, 2], in which one assumes a given and fixed decoder, $\mathbf{D}$, and then derives the best network architecture for that decoder.

Specifically, we will seek to find the set of spike trains, $\mathbf{o}(t)$, that minimizes the loss function

$$L(\mathbf{o}) = \left\langle \|\mathbf{x} - \mathbf{Dr}\|^2 + \mu \|\mathbf{r}\|^2 + \nu \|\mathbf{r}\|_1 \right\rangle,$$

where the average in angular brackets is taken over all possible spike trains. To do so, we will first consider the effect of a spike of neuron n on the instantaneous loss ℓ (S.6). The spike will increase the filtered spike train, $r_n \to r_n + 1$, and thus update the signal estimate according to $\hat{\mathbf{x}} \to \hat{\mathbf{x}} + \mathbf{D}_n$, where $\mathbf{D}_n$ is the n-th column of the decoder matrix $\mathbf{D}$. We can therefore rewrite the objective (S.6) after the firing of the spike as

$$\ell(\text{neuron } n \text{ spiked}) = \|\mathbf{x} - \hat{\mathbf{x}} - \mathbf{D}_n\|^2 + \mu \|\mathbf{r} + \mathbf{e}_n\|^2 + \nu \|\mathbf{r} + \mathbf{e}_n\|_1,$$

where $[\mathbf{e}_n]_j = \delta_{nj}$. We adopt a greedy optimization scheme in which a neuron fires as soon as its spike decreases the loss. Mathematically, this condition yields the expression $\ell(n \text{ spiked}) < \ell(n \text{ did not spike})$, from which we can immediately derive the spiking condition [2, 1],

$$\mathbf{D}_n^\top \mathbf{x} - \mathbf{D}_n^\top \mathbf{Dr} - \mu r_n > \frac{1}{2}(\|\mathbf{D}_n\|_2^2 + \mu + \nu).$$

Notice that all terms on the l.h.s. are time-dependent while all terms on the r.h.s. are constant. We will identify the l.h.s. with the (time-varying) voltages of our neurons, and the r.h.s. with the (constant) thresholds,

$$V_n(t) = \mathbf{D}_n^\top \mathbf{x} - \mathbf{D}_n^\top \mathbf{Dr} - \mu r_n, \tag{S.10}$$

$$T_n = \frac{1}{2}(\|\mathbf{D}_n\|^2 + \mu + \nu). \tag{S.11}$$

We can now compare this equation to the equation of the integrated network voltages, (S.8) and (S.9). Accordingly, to be optimal, a network should have the following connectivity,

$$\mathbf{F} = \mathbf{D}^\top, \tag{S.12}$$

$$\begin{aligned}\mathbf{\Omega} &= -\mathbf{D}^\top\mathbf{D} - \mu\mathbf{I} \\ &= -\mathbf{F}\mathbf{F}^\top - \mu\mathbf{I},\end{aligned} \tag{S.13}$$

where $\mathbf{I}_N$ is the $N \times N$ identity matrix.[4] These connectivities are similar to those of optimal rate networks that are designed to minimize the loss function (S.6) [3,

[4]Note that, since we are now ignoring Dale's law, all synaptic weights can be both excitatory and inhibitory. For illustrative purposes, however, it is often useful to consider the case in which all feedforward weights are excitatory, and hence all lateral weights are inhibitory (in the optimal network). We will consider this toy scenario throughout the SI.

4, 5, 6]. They are quite specific in that the recurrent weights are symmetric and, as shown in the last equation, are directly related to the feedforward weights.

Furthermore, the thresholds are related to the recurrent (and thereby the feedforward connectivities) via (S.11) so that

$$\begin{aligned}\mathbf{T} &= \frac{1}{2}\left(\operatorname{diag}(\mathbf{D}^\top\mathbf{D}) + \mu + \nu\right)\\ &= \frac{1}{2}\left(-\operatorname{diag}(\mathbf{\Omega}) + \nu\right),\end{aligned}$$

where $\mathbf{T}$ is simply the N-dimensional vector of all thresholds. This equation may seem to pose an additional condition on the network architecture. However, as explained in the next section, this condition can be reformulated as a simple constraint on the decoder length.

Two important observations follow from this derivation. First, we note that the spiking condition of a single neuron relies on local information only and does not require the evaluation or knowledge of the full objective function. Indeed, rewriting (S.10) by using the definition of $\hat{\mathbf{x}}$, we obtain

$$V_n(t) = \mathbf{D}_n^\top(\mathbf{x} - \hat{\mathbf{x}}) - \mu r_n, \tag{S.14}$$

so that the voltage reflects both the part of the reconstruction error, $\mathbf{x} - \hat{\mathbf{x}}$, that is projected onto the decoder weights $\mathbf{D}_n$, as well as the quadratic cost term. Accordingly, a spike fired by a neuron is designed to decrease this projected error, and in turn decreases the objective [1]. Biophysically, a depolarization of the membrane voltage (through excitatory inputs) signals an increase in the projected error. A repolarization (through inhibitory inputs or the self-reset after a spike) signals an elimination in the projected error. This 'balancing' of the coding errors is equivalent to the balance of excitation and inhibition.

A second observation is that the optimal architecture deviates from the error-driven architecture from Section 3, as the feedforward weights $\mathbf{F}$ are not necessarily equal to the transpose of the decoder $\mathbf{D}^\top$. In turn, we obtain one further necessary condition for optimality, which is

Condition 3: $\mathbf{F} = \mathbf{D}^\top$

meaning that in the optimal architecture, the feedforward weights have to become equal to the decoder. Since the decoder is not a priori given for a general network such as the one in Section 2, we need one additional constraint for the decoder. The next section will explain how this constraint comes about.

Importantly, even if the feedforward weights, $\mathbf{F}$, deviate from the decoder weights, $\mathbf{D}^\top$, the voltages of the neurons will still reflect projections of the reconstruction error. Furthermore, as long as this deviation is not too large,[5], the spike fired by a neuron will decrease the reconstruction error.

[5] We consider a 'large' deviation one in which the dot product of at least one set of feedforward and decoder weights becomes too small or becomes negative, $\mathbf{F}_i^\top\mathbf{D}_i < \epsilon$.

5 The optimal decoder

The loss function (S.4) defines the quality of a particular reconstruction, $\hat{\mathbf{x}} = \mathbf{Dr}$. A straightforward way to find the best connectivity in the network is therefore to simply optimize the loss function with respect to the decoder matrix $\mathbf{D}$. In turn, the connectivity can be computed using the equations (S.12) and (S.13) derived in the previous section. The key problem with this approach is that it is not biophysical, since the decoder $\mathbf{D}$ does not have a physical basis in the network. Nonetheless, it will be useful to go through this exercise, as it ties to some of the efficient coding literature of the past (e.g. [18] and related studies), and will provide a useful foundation for the derivation of the actual learning rules.

5.1 Solution for arbitrary networks

In short, we want to determine the best possible decoder $\mathbf{D}$ for a given network, i.e., minimize

$$L(D) = \left\langle \|\mathbf{x} - \mathbf{Dr}\|^2 \right\rangle ,$$

where $\mathbf{r}$ is the filtered spike train output of the network. Note that we left out the cost terms since they do not depend on the decoder and therefore have no influence on the solution. Taking the derivative of the above loss function with respect to $\mathbf{D}$, we have

$$\frac{\partial L}{\partial \mathbf{D}} = -2 \left\langle (\mathbf{x} - \hat{\mathbf{x}})\mathbf{r}^\top \right\rangle ,$$

which is a learning rule for the decoder. We can determine the fixed point of this learning rule by setting it to zero, which yields

$$\mathbf{D} = \left\langle \mathbf{x}\mathbf{r}^\top \right\rangle \left\langle \mathbf{r}\mathbf{r}^\top \right\rangle^{-1} .$$

(Note that this solution is the well-known solution to the linear regression problem, where $\mathbf{r}$ are the regressors, $\mathbf{x}$ are the regressands , and $\mathbf{D}$ are the coefficients.) We can employ this formula to find the respective optimal decoder for any network, i.e., for arbitrary feedforward or recurrent connectivities. Indeed, we use this formula in Figure 2 in the main text, where we compare the reconstructed signal with the input signal both for the unlearnt network and for the learnt network.

5.2 Solution under length constraints

Section 4 showed that the thresholds, T_n, in the optimal network are intimately linked to the L2 norm of the decoder weights. If we consider the thresholds as fixed, then we can re-interpret (S.11) as imposing a constraint on the length of the (optimal) decoder,

$$\|\mathbf{D}_n\|_2^2 = 2T_n - \mu - \nu =: a_n. \tag{S.15}$$

To phrase the corresponding optimization problem, we can use a set of Lagrangian multipliers λ_n, and simply add these length constraints to the mean

square error of the reconstruction to obtain the modified loss function

$$L = \left\langle \|\mathbf{x} - \mathbf{D}\mathbf{r}\|^2 \right\rangle + \sum_{n=1}^{N_E} \lambda_n \left(\|\mathbf{D}_n\|^2 - a_n \right).$$

Taking the derivative of the loss with respect to $\mathbf{D}_n$ (and remembering that $\hat{\mathbf{x}} = \sum_n \mathbf{D}_n r_n$) yields

$$\frac{\partial L}{\partial \mathbf{D}_n} = -2 \left\langle (\mathbf{x} - \hat{\mathbf{x}}) r_n \right\rangle + 2\lambda_n \mathbf{D}_n.$$

In turn, we can set the derivative to zero to find the minimum of the loss function. An insightful *implicit* solution is found by writing

$$\mathbf{D}_n = \frac{1}{\lambda_n} \left\langle (\mathbf{x} - \hat{\mathbf{x}}) r_n \right\rangle, \tag{S.16}$$

and illustrates that the decoder will generally align with the direction of the largest reconstruction errors whenever the neuron fires strongly. An explicit solution can be found as well, corresponding to a penalized least square solution, and is given by

$$\mathbf{D} = \left\langle \mathbf{x}\mathbf{r}^\top \right\rangle \left\langle \mathbf{r}\mathbf{r}^\top + \mathbf{\Lambda} \right\rangle^{-1}.$$

where $\mathbf{\Lambda}$ is a diagonal matrix whose entries are the Lagrangian constraints, λ_n.

5.3 The optimal decoder for the optimal network architecture

In Section 4, we found the optimal network architecture for a given decoder, while in this section, we discussed the optimal decoder for a given network architecture. Ideally, of course, we want to perform both optimizations at the same time. We therefore require our networks to perform a double-minimization with respect to both the spike times $\mathbf{o}$ as well as with respect to the decoder $\mathbf{D}$,

$$L^* = \min_{\mathbf{o},\mathbf{D}} \left\langle \|\mathbf{x} - \mathbf{D}\mathbf{r}\|^2 + \mu \|\mathbf{r}\|^2 + \nu \|\mathbf{r}\|_1 \right\rangle \text{ s.t. } \|\mathbf{D}_n\|_2^2 = 2T_n - \mu - \nu, \tag{S.17}$$

where the constraint on the decoder length arises from (S.11).

This insight leads us to another condition on the learning of the feedforward and recurrent weights. In condition 1–3, we made use of an (unknown) decoder matrix $\mathbf{D}$. Our fourth condition states that this matrix, at the end of learning should converge to

Condition 4: $\mathbf{D} = \mathbf{D}^*$ where $\mathbf{D}^*$ realizes the minimum of (S.17)

6 The four conditions of learning

We will describe two ways of learning the connectivity, one based on a balancing of input currents (Sections 7 and 8), and one based on minimizing voltage fluctuations (Sections 9 and 10). To see through these derivations, we will first explain the overall logic behind the learning of the recurrent and feedforward weights, which is identical for both derivations. Recapitulating the insights from Sections 3–5, we obtain four conditions on learning, two for the recurrent and two for the feedforward weights.

6.1 Condition 1: Spike-by-spike balance

The membrane voltage of each neuron should remain close to zero, i.e., its resting potential. We can interpret this to mean that the membrane voltage fluctuations should be minimized or bounded as tightly as possible. Accordingly, any deviation from rest caused by the feedforward inputs must be immediately eliminated by the recurrent inputs. In other words, the feedforward and recurrent inputs into each cell need to balance each other on short time-scales. As a consequence, any excitatory input into the cells must be quickly canceled by an inhibitory input of equal size, a condition known as tight EI balance, or otherwise it would cause a spike and a self-reset of the voltage. Since the recurrent inputs are given by the spikes of other neurons, we also refer to this balance as spike-by-spike balance.

6.2 Condition 2: Recurrent weights need to be $\mathbf{\Omega} = -\mathbf{FD}$

The recurrent connectivity should be factorizable into the form $\mathbf{\Omega} = -\mathbf{FD}$ where $\mathbf{F}$ is the matrix of feedforward weights and $\mathbf{D}$ is an a priori unknown decoder matrix.[6] As a consequence, the membrane voltage of each neuron can be interpreted as a *projection of the reconstruction error*, ε_n, which we refer to as 'error-driven coding.' Indeed, the recovery of the reconstruction error in the membrane voltages is a key ingredient of the optimal network, see equation (S.14). While the decoder matrix, $\mathbf{D}$, is unspecified at this point, not all matrices $\mathbf{D}$ will allow a network to fulfill condition (1), as well. As a consequence, the target $\mathbf{\Omega} = -\mathbf{FD}$ consists of a large, if unspecified, set of possible matrices, and 'error-driven coding' can be achieved by a large set of networks. Once $\mathbf{\Omega}$ has converged, the decoder $\mathbf{D}$ can, in principle, be derived by solving the equation $\mathbf{\Omega} = -\mathbf{FD}$ for $\mathbf{D}$.

6.3 Interlude: the importance of quadratic costs

The 'error-driven coding' architecture achieves the primary objective, i.e. representing the signal $\mathbf{x}(t)$, which can now be read out via the decoder $\mathbf{D}$ (once the decoder has been determined). Moreover, the voltages of the neurons now represent part of the global coding error, or, more generally, the loss function.

However, even though the loss function is now represented within the network, it is not yet minimized. More specifically, the efficiency of the representa-

[6] We note that the decoder $\mathbf{D}$ is a central conceptual tool in the formulation of the learning rules. This decoder is not an explicit biophysical quantity of the network, and is initially simply an unknown matrix $\mathbf{D}$ of size $M \times N$.

tion depends strongly on the exact choice of the feedforward weights. There are two problems. First, if the feedforward weights do not cover some part of the input space (as in Figure 2, central column), then the reconstruction cost can still be high in that part of the space. Second, even if the feedforward weights cover the whole space, so that $\hat{\mathbf{x}} \approx \mathbf{x}$ everywhere, the particular spiking code chosen by the system can still be inefficient from a practical point of view: since we have not considered any cost terms, neurons could fire at very high rates in order to properly represent the signal. To find a better distribution of the feedforward weights, we therefore need to first re-introduce the L2 cost term, i.e., the cost term that severely punishes high firing of individual neurons.

In the presence of an L2 cost, we know the form of the optimal recurrent connectivity from section 4. Adapted to the error-driven coding architecture, the recurrent weights should therefore converge to $\mathbf{\Omega} = -\mathbf{FD} - \mu\mathbf{I}$.

6.4 Condition 3: Feedforward weights need to mimic D

The network architecture that we have derived so far still deviates from the optimal architecture, since $\mathbf{F}$ and $\mathbf{D}^\top$ are not necessarily the same matrices. Accordingly, we need to somehow make sure that the feedforward weights $\mathbf{F}$ align with the decoder $\mathbf{D}^\top$.

This update of the feedforward connections should happen at a slower time scale than recurrent learning, to ensure that the equation $\mathbf{\Omega} = -\mathbf{FD} - \mu\mathbf{I}$ is approximately preserved.

6.5 Condition 4: Decoder needs to minimize the loss

To make sure that the network as a whole represents the input signals properly, the feedforward weights need to properly span the space of input signals. If, for instance, the feedforward weights of all neurons were identical, the network could at most represent the one-dimensional space spanned by these feedforward weights—a pathological and uninteresting solution. We can make sure that the signal space is properly covered if both $\mathbf{D}$ and $\mathbf{F}$ converge to the global optimum $\mathbf{D}^*$ of (S.17).

6.6 A plan of action for learning all the weights

These conditions suggest a specific program for learning the synaptic weights. First, starting from random feedforward and recurrent weights, we need to learn a balanced system in which the recurrent weights converge to a low-rank solution, $\mathbf{\Omega} \to -\mathbf{FD} + \mu\mathbf{I}$. In a second step, we can then aim to tighten the balance between excitatory and inhibitory currents by aligning $\mathbf{D}$ and $\mathbf{F}$ such that both converge to the global optimum, $\mathbf{D}^*$.

In the next sections, we follow this program through for the current-based learning rules and then the voltage-based learning rules. Only in the latter case will we provide convergence proofs and also explain the full generalization to L1 costs and non-whitened inputs.

7 Recurrent weights: current-based learning

In this section, we will explain how the current-based learning rules achieve error-driven coding, or conditions 1 and 2. The current-based learning rules are mathematically simpler, yet biophysically less plausible than the voltage-based rules. To ease the presentation, this section does not consider the most general scenario (with both L1 and L2 costs and correct scaling), which we will revisit in section 9, where the voltage-based learning rules are derived.

7.1 Condition 1: Recurrent weights learn to balance

The shortest possible time-scale at which a single spiking neuron can be balanced is limited by the interval between any two consecutive *population spikes*, i.e. spikes emitted by any neuron in the population. We will refer to this interval as a population interspike interval (pISI), in contrast to the standard interspike interval (ISI) that is defined for two consecutive spikes of the *same* neuron.

We illustrate this idea in Figure 1C in the main text. Here a cell receives excitatory feedforward inputs and inhibitory spikes from three pre-synaptic neurons. Between the second and the third spike (gray area) the cell integrates its feedforward input currents and depolarizes its membrane voltage. The arrival of the second inhibitory spike (red) then causes a hyperpolarization of the membrane potential (see voltage trace in middle panels). In the balanced case (left column) the hyperpolarization due to the inhibitory spike from the red neuron perfectly cancels the depolarization through the excitatory feedforward connections (gray area).

To understand how to balance a single cell on such a short time-scale, we rewrite the membrane potential as a sum over spikes. We index the spikes in the network by the time of their occurrence, writing $t_1, t_2, \ldots$ for the successive spike times of the population. We then introduce a second index in order to identify which neuron fired a particular spike, writing $k(i)$ to indicate that the i-th spike, t_i, was fired by the k-th neuron. With this notation in mind, let us define the integral over the input signal $\mathbf{c}(t)$ in the interval between two consecutive population spikes at time t_{i-1} and t_i:

$$\mathbf{g}(t_i) := \int_{t_{i-1}}^{t_i} d\tau\, \mathbf{c}(\tau) e^{-\lambda(t_i-\tau)}.$$

We can then write the membrane voltage V_n at time t_i (i.e., at the time of the i-th spike) as (confer (S.8))

$$\begin{aligned} V_n(t_i) &= \mathbf{F}_n^\top \mathbf{x}(t_i) + \boldsymbol{\Omega}_n^\top \mathbf{r}(t_i) \\ &= \mathbf{F}_n^\top \int_{-\infty}^{t_i} d\tau\, \mathbf{c}(\tau) e^{-\lambda(t_i-\tau)} + \boldsymbol{\Omega}_n^\top \int_{-\infty}^{t_i} d\tau\, \mathbf{o}(\tau) e^{-\lambda(t_i-\tau)} \\ &= \sum_{j\le i} \mathbf{F}_n^\top \mathbf{g}(t_j) e^{-\lambda(t_i-t_j)} + \sum_{j\le i} \Omega_{nk(j)} e^{-\lambda(t_i-t_j)} \\ &= \sum_{i\le j} (\mathbf{F}_n^\top \mathbf{g}(t_j) + \Omega_{nk(j)}) e^{-\lambda(t_i-t_j)}, \end{aligned}$$

where $k(j)$ denotes the index of the neuron spiking at time t_j, as explained above. Here $\mathbf{F}_n\mathbf{g}(t_j)$ corresponds to the accumulated excitatory current during the pISI before the j-th spike, i.e., the total charge transfer. In turn, $\Omega_{nk(j)}$ corresponds to the immediate inhibitory charge transfer caused by the j-th spike itself. The network is perfectly balanced on the shortest time scale if these two opposing charge transfers cancel exactly. In more practical terms, the network is balanced on the shortest time scale if the (squared) net charge transfer, $\left(\mathbf{F}_n^\top \mathbf{g}(t_j) + \Omega_{nk(j)}\right)^2$, is as small as possible.

We hence concentrate on minimizing the objective

$$L = \sum_{n,i} \left(\mathbf{F}_n^\top \mathbf{g}(t_i) + \Omega_{nk(i)}\right)^2 ,$$

where the sum runs over both neurons, n, and spike times, i. We can minimize this objective by updating the recurrent synaptic weights after each spike in a greedy manner,

$$\Delta\Omega_{nk}(t) \propto \begin{cases} -\mathbf{F}_n^\top \mathbf{g}(t) - \Omega_{nk}(t) & \text{when neuron } k \text{ spikes,} \\ 0 & \text{otherwise.} \end{cases} \tag{S.22}$$

This rule has a rather intuitive meaning, as explained in the main paper and illustrated in Fig 1C: if the excitation a neuron receives in the last pISI is higher than the subsequent lateral inhibition, inhibition is strengthened, and vice versa[7]. Importantly, the learning rule relies only on local information, i.e. on quantities that are available to the neuron that needs to update its synapses.

7.2 Condition 2: Spike-by-spike balance yields error-driven coding

By using the above learning rule for the recurrent weights, we can establish spike-by-spike balance—condition (1) in Section 3—for every single neuron $n = 1 \dots N$. We will now show that condition (2) comes out as a by-product of this learning rule, yielding the error-driven coding architecture.

The proof is straightforward. We simply investigate the fixed points of the learning rule (S.22), i.e. all points for which the mean update is zero, $\langle \Delta\mathbf{\Omega} \rangle = 0$. We obtain

$$\Omega_{nk} = -\left\langle \mathbf{F}_n^\top \mathbf{g} \right\rangle_{k \text{ spikes}} = -\mathbf{F}_n^\top \langle \mathbf{g} \rangle_{k \text{ spikes}} ,$$

where the brackets denote an average taken over all the spike-times of neuron k. This formula has two interesting consequences. First, the strength of the inhibitory synapse from neuron k to neuron n equals the average excitatory feedforward current that neuron n receives in the pISI before a spike of neuron k. Thus, all neurons in the population cooperate to keep the voltage of the post-synaptic cell as constant as possible. Second, the fixed points for the recurrent

[7]Note once more that, for illustrative purposes, we here suppose that the recurrent weights are inhibitory and all feedforward weights are excitatory. We use this simplified picture for the rest of the SI. In the case described here, in which the network violates Dale's law, feedforward and recurrent weights can have both positive as well as negative signs.

weights can be written as $\Omega_{nk} = -\mathbf{F}_n^\top \mathbf{D}_k$ where[8]

$$\mathbf{D}_k = \langle \mathbf{g} \rangle_{k \text{ spikes}} . \tag{S.23}$$

We note that this is the desired low-rank factorization for "error-driven coding", i.e., $\boldsymbol{\Omega} \to -\mathbf{FD}$. In this regime the membrane voltage of each cell tracks a projection of the error, and so the network fulfills condition (2) in section 3.

To summarize, we derived a simple learning rule for the recurrent connections from the principle that each recurrently fired spike should balance the feedforward input of its respective postsynaptic cells. This rule seeks to balance the network on the shortest possible time scale and thereby yields the desired low-rank factorization of the recurrent weights which is important for error-driven coding. Furthermore, we have derived an explicit formula for the decoder. Hence, even though the decoder was initially unknown, and even though the decoder does not have a direct biophysical manifestation, it can be computed through biophysical quantities, namely, the input signal sampled at the spikes of the different neurons.

[8]Please note that $\mathbf{F}_k$ corresponds to the k-th *row* of matrix $\mathbf{F}$ while $\mathbf{D}_k$ corresponds to the k-th *column* of matrix $\mathbf{D}$. Nonetheless, all vectors in the SI, including these two, are assumed to be column vectors.

8 Feedforward weights: current-based learning

In this section, we will explain how the feedforward weights can be learnt within the current-based framework, and we will focus on conditions (3) and (4) explained in Section 6. As also explaind in that section, the quadratic costs become important in order to specify exactly which feedforward weights we consider optimal, and we will therefore first specify how the introduction of quadratic costs modifies the recurrent learning rules. (We note that the current-based learning of the feedforward weights is not explained in the main text. We include it here for the sake of completeness.)

8.1 Quadratic costs modify recurrent learning

In the presence of an L2 cost, we know the form of the optimal recurrent connectivity from section 4. Adapted to the error-driven coding architecture, the recurrent weights should therefore converge to $\mathbf{\Omega} = -\mathbf{FD} - \mu\mathbf{I}$. We can achieve this new fixed point of the recurrent learning rule (S.22) by introducing a small regularization term, $\mu\delta_{ij}$, so that

$$\Delta\Omega_{nk}(t) \propto \begin{cases} -\mathbf{F}_n^\top \mathbf{g}(t) - \Omega_{nk}(t) - \mu\delta_{nk} & \text{when neuron } k \text{ spikes,} \\ 0 & \text{otherwise.} \end{cases} \tag{S.24}$$

Importantly, the learning rule will still seek to balance the system as tightly as possible given the extra constraints. Following the logic of the previous section, one can see that $\mathbf{\Omega}$ will converge to the desired fixed point. After convergence of the recurrent weights to $\mathbf{\Omega} = -\mathbf{FD} - \mu\mathbf{I}$, the membrane potential of each neuron can be written as

$$\mathbf{V} = \mathbf{F}(\mathbf{x} - \mathbf{Dr}) - \mu\mathbf{r}.$$

As a side note, we point out that the introduction of the cost term does not alter the definition of the decoder (S.23).

8.2 Condition 3: Feedforward weights learn to mimic the decoder

The derivation of the optimal network, section 4, suggests that $\mathbf{F}$ should eventually align with $\mathbf{D}^\top$, as explained in condition (3) in section 3. Ideally, one would therefore want an update rule of the form $\Delta\mathbf{F} \propto \mathbf{D}^\top - \mathbf{F}$, which would move the feedforward weights towards the decoder $\mathbf{D}^\top$. While $\mathbf{D}$ can be inferred from the recurrent connectivity by solving $\mathbf{\Omega} = -\mathbf{FD} - \mu\mathbf{I}$ for $\mathbf{D}$, it is not an explicit quantity in the network, and the above learning rule is therefore biophysically unrealistic. However, using the fixed-point equation for the decoder, (S.23), we can replace $\mathbf{D}$ to obtain a local, biophysical rule

$$\Delta\mathbf{F}_n(t) \propto \begin{cases} \mathbf{g}(t) - \mathbf{F}_n(t) & \text{when neuron } k \text{ spikes,} \\ 0 & \text{otherwise.} \end{cases}$$

If the learning of the feedforward connectivity occurs more slowly than the learning of the recurrent connectivity, then all fixed points of the feedforward network connectivity will fulfill $\mathbf{F}_n = \langle\mathbf{g}\rangle_{n \text{ spikes}} = \mathbf{D}_n$ and hence $\mathbf{F} \to \mathbf{D}^\top$

as desired. From a biophysical perspective the first term in the learning rule, $\langle \mathbf{g} \rangle_{n \text{ spikes}}$, corresponds to the average input signal integrated before a spike of neuron n. Such a signal could be computed in the presynaptic terminal, for instance, which, given the complex machinery of synaptic plasticity is well within the realm of possibilities.

We make two observations about the feedforward rule. First, the rule will only change the feedforward weights if a postsynaptic spike (of neuron n) coincides with a previous presynaptic input (the integrated input signal up to the time of the postsynaptic spike). In other words, this learning rule corresponds to the causal part of the standard STDP-rule. Second, a neuron that never spikes will not change its feedforward weight. This latter scenario is problematic since the neuron is then essentially lost to the network. However, it can be avoided either by introducing a noise term in the learning rule, or by lowering the neuron's threshold. We used this latter solution to overcome this problem in the initial stages of learning for Figure 4–8, see also Section 14.

8.3 Condition 4: Learning rules minimize loss function

While the feedforward learning rule shapes the connectivity into the desired form (section 4), it is not a priori clear whether these changes also help to minimize the loss function, (S.17), which was our fourth condition on learning. We will now show that the learning rule derived in the previous section achieves exactly that. To do so, we will investigate how the learning rules affect the (average) voltages, since the voltages are directly linked to the reconstruction errors and thereby the loss function. To keep things simple, we will assume that the recurrent connectivity is already learnt, and we will write $\Delta \mathbf{F} = \lambda(\mathbf{D}^\top - \mathbf{F})$ for the feedforward update, where λ is a small feedforward learning rate. Let $\Delta \mathbf{r}$ be the corresponding change in firing rate. The resulting change in the voltage will then—on average—be proportional to

$$\begin{aligned}
\langle \Delta \mathbf{V} \rangle &= \langle \Delta \mathbf{F}(\mathbf{x} - \mathbf{D}\mathbf{r}) + \boldsymbol{\Omega} \Delta \mathbf{r} \rangle \\
&= \lambda \langle (\mathbf{D}^\top - \mathbf{F})(\mathbf{x} - \mathbf{D}\mathbf{r}) \rangle + \langle \boldsymbol{\Omega} \Delta \mathbf{r} \rangle \\
&= \lambda \langle \mathbf{D}^\top (\mathbf{x} - \mathbf{D}\mathbf{r}) - \mu \mathbf{r} \rangle - \lambda \langle \mathbf{F}(\mathbf{x} - \mathbf{D}\mathbf{r}) - \mu \mathbf{r} \rangle + \langle \boldsymbol{\Omega} \Delta \mathbf{r} \rangle \\
&= \lambda \langle \mathbf{D}^\top (\mathbf{x} - \mathbf{D}\mathbf{r}) - \mu \mathbf{r} \rangle - \lambda \langle \mathbf{V} \rangle + \langle \boldsymbol{\Omega} \Delta \mathbf{r} \rangle .
\end{aligned}$$

Since the learning of $\mathbf{F}$ happens on a much slower time-scale than the learning of the recurrent weights, both the l.h.s, i.e. the average voltage difference, and the second term on the r.h.s., i.e., the average voltage, will be close to zero, as the network remains in a tightly balanced state throughout the learning of the feedforward weights. As a consequence the change in firing rates approximately respects the following equation

$$\begin{aligned}
\langle \boldsymbol{\Omega} \Delta \mathbf{r} \rangle &\approx -\lambda \langle \mathbf{D}^\top (\mathbf{x} - \mathbf{D}\mathbf{r}) - \mu \mathbf{r} \rangle, \\
&= \lambda \frac{\partial L}{\partial \mathbf{r}}
\end{aligned}$$

where L is the averaged loss function (S.4) in the absence of the linear cost term. As the network is in the balanced state, we expect the symmetric part of

$\boldsymbol{\Omega}$ to be negative-definite, that is for all $\mathbf{v} \neq 0$, $\mathbf{v}^\top \boldsymbol{\Omega} \mathbf{v} < 0$. As a consequence

$$
\begin{aligned}
-\langle \Delta \mathbf{r}^\top \rangle \cdot \frac{\partial L}{\partial \mathbf{r}} &= -\frac{1}{\lambda} \langle \Delta \mathbf{r}^\top \rangle \cdot \boldsymbol{\Omega} \langle \Delta \mathbf{r}^\top \rangle \\
&> 0.
\end{aligned}
$$

In other words, the change in instantaneous firing rate of the network will have positive dot product with the antigradient of the loss function, thus minimising it.

8.4 Conclusions and biological realism reconsidered

To summarize, in this section we illustrated how an STDP-like learning rule for the feedforward connections in conjunction with a recurrent learning rule that seeks to tightly balance excitatory and inhibitory inputs, leads to a network architecture that optimizes the average loss, (S.4), and thereby produces an efficient spike code of the input signals. The derived learning rules are local, in that they only require knowledge of quantities that are available to the neurons.

There are several issues that we did not consider so far. First, we did not study linear costs or non-whitenend input distributions. Second, even though the learning rules are local, their biological plausibility may still be questioned, since the rules require the integration of input currents between successive spikes of the population. While it cannot be ruled out that actual neurons (or synapses) do keep track of these quantities, it has not been observed, either. In the next section, we address these concerns and derive learning rules based on the voltages of the neurons. We furthermore consider the extension to linear costs, non-whitened inputs, correct scaling and, finally, the full EI network.

9 Recurrent weights: Voltage-based learning

In this section, we will explain how the voltage-based learning rules achieve error-driven coding, or conditions 1 and 2. These are the recurrent learning rules that underlie the simulations in the main text. The derivation in this section closely follows the spirit of Section 7, and includes a convergence proof.

9.1 Condition 1: Recurrent weights learn to minimize voltage fluctuations

As in Section 7, we start by considering the learning of the recurrent synapses without costs. The target of learning is then a balanced network with recurrent weights $\mathbf{\Omega} = -\mathbf{FD}$. In the last section we showed that it is enough to seek a balanced state in order to reach both properties. In the same spirit, we first establish a suitable and practical measure of membrane voltage fluctuations, derive learning rules for the recurrent weights that minimize those fluctuations and then show that the network will converge to a low-rank configuration $\mathbf{\Omega} \to -\mathbf{FD}$.

A particularly straightforward way of measuring voltage fluctuations is the temporal average of the squared voltage deviations from rest $V_0 = 0$, i.e.,

$$L = \left\langle \|\mathbf{V}(t)\|^2 \right\rangle_t .$$

However, evaluating the exact voltage deviations requires a precise tracking of the membrane voltage at all times, and may thus be infeasible for real neurons. Inspired by the insights from the previous section, we start with the presumption that learning occurs only during the presence of a presynaptic spike. We can then reduce the problem to minimizing the deviations of the membrane voltages around the time of a presynaptic spike. In the optimal network, the membrane voltage of a spiking neuron jumps from the threshold, T, *before* the spike to the reset, $-T$, *after* the spike. Similarly, to achieve tight balance the membrane voltage of all other neurons should ideally jump from $+V$ before a presynaptic spike to $-V$ after the spike. This motivates the following spike-based measure of the membrane voltage fluctations,

$$L = \left\langle \left\| \frac{1}{2}\left(\mathbf{V}^{\text{before}}(t_j) + \mathbf{V}^{\text{after}}(t_j)\right) \right\|^2 \right\rangle_{\text{spikes}} \tag{S.25}$$

where t_j is the time of the j-th spike in the population and $\langle \cdot \rangle_{\text{spikes}}$ denotes the expectation value over those spikes[9]. The superscripts "before" and "after" refer to the voltage values immediately before and after a spike. The recurrent weights enter (S.25) through their effect on the post-spike membrane potential,

$$\mathbf{V}^{\text{after}}(t_j) = \mathbf{V}^{\text{before}}(t_j) + \mathbf{\Omega}\mathbf{e}_{k(j)},$$

where $k(j)$ is again the index of the neuron that spikes at time t_j and $\mathbf{e}_{k(j)}$ is a unit vector with zero entries except at position $k(j)$. The introduction of

[9]Note that in practice, i.e., in numerical simulations, we replace this expectation value with a moving sum over a sufficiently large number of spikes. More precisely, we set $\langle x \rangle_{\text{spikes}} = \frac{1}{J}\sum_{j=1}^{J} x(t_j)$ where t_j is the spike-time of the j-th spike in the past (relative to the current time t).

$\mathbf{e}_{k(j)}$ is a bit cumbersome but useful: it allows, for example, to write the relation between the instantaneous rates of the whole population before and after a spike of neuron k at time t_j as a simple vector equation,

$$\mathbf{r}^{\text{after}}(t_j) = \mathbf{r}^{\text{before}}(t_j) + \mathbf{e}_{k(j)}.$$

The deviation at time t_j is thus

$$\begin{aligned} L_j &= \left\| \frac{1}{2} \left(\mathbf{V}^{\text{before}}(t_j) + \mathbf{V}^{\text{after}}(t_j) \right) \right\|^2 \\ &= \left\| \mathbf{V}^{\text{before}}(t_j) + \tfrac{1}{2} \boldsymbol{\Omega} \mathbf{e}_{k(j)} \right\|^2 . \end{aligned}$$

To minimize the voltage deviations, we perform a greedy optimization every time a spike was fired by one of the neurons in the network,

$$\Delta\boldsymbol{\Omega}(t_j) \propto -\frac{\partial L_j}{\partial \boldsymbol{\Omega}} \propto -\left(2\mathbf{V}^{\text{before}}(t_j) + \boldsymbol{\Omega}\mathbf{e}_{k(j)} \right) \mathbf{e}_{k(j)}^{\top}. \tag{S.26}$$

More explicitely, the weight Ω_{nk} is updated at every spike of the presynaptic neuron $k = k(j)$ such that the deviation of the postsynaptic membrane voltage from rest is minimized,

$$\Delta\Omega_{nk}(t_j) \propto \begin{cases} -2V_n^{\text{before}}(t_j) - \Omega_{nk} & \text{if } k \text{ spiked,} \\ 0 & \text{otherwise.} \end{cases} \tag{S.27}$$

This learning rule, when rewritten as a differential equation, is exactly equivalent to Equation (5) in the main text. (The equivalency can be most easily seen by integrating Equation (5) from the main text over time, and by replacing the index n by the index i.) The rule differs from the one in the last section mainly in the first term on the r.h.s.: instead of compensating for the integrated feedforward current during the last pISI, the synapses here learn to compensate for the deviation of the membrane voltage from rest.

9.2 Condition 2: Recurrent weights reach error-driven-coding architecture

In this section we show that the fixed-points (i.e. the points at which the mean change in the recurrent weights is zero) of the learning rule (S.27) are of the desired low-rank configuration $\boldsymbol{\Omega} \to -\mathbf{FD}$. In the next section we will then prove that the system will globally converge to one of these fixed-points under mild assumptions.

Mathematically, the fixed-points are defined as those recurrent weights for which $\langle \Delta\boldsymbol{\Omega} \rangle_{\text{spikes}} = 0$. All quantities below, such as $\mathbf{V}(t_j)$, $\mathbf{x}(t_j)$, etc., are to be understood as immediately *before* a spike, and we hence drop the superscript "before" for the rest of the SI, as well as the explicit reference to the spike time,

t_j, for ease of notation[10]. The learning rule, (S.26), can then be rewritten as

$$\begin{aligned}\Delta\boldsymbol{\Omega} &\propto -2\mathbf{V}\mathbf{e}_{k(j)}^\top - \boldsymbol{\Omega}\mathbf{e}_{k(j)}\mathbf{e}_{k(j)}^\top \\ &\overset{(S.8)}{=} -2(\mathbf{F}\mathbf{x}+\boldsymbol{\Omega}\mathbf{r})\mathbf{e}_{k(j)}^\top - \boldsymbol{\Omega}\mathbf{e}_{k(j)}\mathbf{e}_{k(j)}^\top \\ &= -2\mathbf{F}\mathbf{x}\mathbf{e}_{k(j)}^\top - \boldsymbol{\Omega}\left(2\mathbf{r}+\mathbf{e}_{k(j)}\right)\mathbf{e}_{k(j)}^\top . \end{aligned} \tag{S.28}$$

To investigate the fixed points, we need to study the effect of applying this learning rule repeatedly, i.e., over many spike times t_j. From (S.28) and the fixed-point condition $\langle\Delta\boldsymbol{\Omega}\rangle_{\text{spikes}} = 0$ we obtain the defining property of the fixed points of the recurrent weights,

$$2\mathbf{F}\left\langle \mathbf{x}\mathbf{e}_{k(j)}^\top \right\rangle_{\text{spikes}} = -\boldsymbol{\Omega}\left\langle \left(2\mathbf{r}+\mathbf{e}_{k(j)}\right)\mathbf{e}_{k(j)}^\top \right\rangle_{\text{spikes}} .$$

Under the mild condition that the sum on the r.h.s. has full rank we can directly infer that any fixed point of $\boldsymbol{\Omega}$ is of the form $-\mathbf{F}\mathbf{D}$ where $\mathbf{D}$ is (implicitly) defined by

$$2\left\langle \mathbf{x}\mathbf{e}_{k(j)}^\top \right\rangle_{\text{spikes}} = \mathbf{D}\left\langle \left(2\mathbf{r}+\mathbf{e}_{k(j)}\right)\mathbf{e}_{k(j)}^\top \right\rangle_{\text{spikes}} . \tag{S.29}$$

While the matrix $\mathbf{D}$ can be interpreted as a linear decoder, which one could use to reconstruct the input signal from the spike trains, it is not explicitly realized within the network, since it is merged into the recurrent weights and arises dynamically through learning. In other words, the decoder is not defined upfront but the recurrent connectivity converges to a low-rank factorization from which an external observer can read off the linear decoder.

Note that (S.29) is a matrix equation with dimensions $M \times N$. To understand the exact nature of the arising decoder $\mathbf{D}$ it is instructive to look at each element i, n individually,

$$\begin{aligned} 2\left\langle x_i\delta_{n,k(j)}\right\rangle_{\text{spikes}} &= \mathbf{D}_i^\top \left\langle \left(2\mathbf{r}+\mathbf{e}_{k(j)}\right)\delta_{n,k(j)}\right\rangle_{\text{spikes}} , \\ \Leftrightarrow 2\left\langle x_i\right\rangle_{\text{n spikes}} &= \mathbf{D}_i^\top \left\langle 2\mathbf{r}+\mathbf{e}_n\right\rangle_{\text{n spikes}} , \end{aligned}$$

where $\langle\cdot\rangle_{\text{n spikes}}$ is simply an average over all the spikes of neuron n. Using the definition for the readout, (S.3), we obtain

$$\begin{aligned} \Leftrightarrow 2\left\langle x_i\right\rangle_{\text{n spikes}} &= 2\left\langle \hat{x}_i\right\rangle_{\text{n spikes}} + D_{in}, \\ \Leftrightarrow D_{in} &= 2\left\langle x_i - \hat{x}_i\right\rangle_{\text{n spikes}} . \end{aligned} \tag{S.30}$$

Accordingly, the elements of the decoder are aligned with the reconstruction errors at the time of a spike. During learning, the optimal decoder will therefore move in directions with the largest error and hence will aim to cover as best as possible the signal space.[11]

The resulting relation for the decoder is essentially equivalent to the constrained optimal decoder derived in section 5, up to a scaling parameter, which we will consider further down. The minimum of the constraint loss function was found as (S.16)

$$D_{in}^* \propto \left\langle (x_i - \hat{x}_i) r_n \right\rangle_t .$$

Accordingly, the weighting of the error by the instantaneous firing rate mirrors the weighting of the error by the spikes in (S.30).

[10] We note that the input signal $\mathbf{x}(t_j)$ does not jump at the time t_j of the presynaptic spike, hence the distinction between before and after is irrelevant for this quantity.

[11] Note that (S.30) is a self-consistency relation since $\mathbf{D}$ is also part of $\hat{x}_i$.

9.3 Condition 2: Convergence proof

In the last subsection we derived that all fixed points of the recurrent weights are of the form $-\mathbf{FD}$, but these fixed points might be unstable. We here prove their stability by showing that any spiking network with bounded membrane voltages will converge to the desired low-rank factorization.

To this end we split the recurrent weights $\mathbf{\Omega}$ into a part that can be described by a low-rank factorization, $\mathbf{\Omega}_F$, and a residual part, $\mathbf{\Omega}_\perp$. The low-rank part, $\mathbf{\Omega}_F$, can be described as the projection of $\mathbf{\Omega}$ onto the image of $\mathbf{F}$, i.e., $\mathbf{\Omega}_F = \mathbf{F}\mathbf{F}^+\mathbf{\Omega}$, where the superscript "+" denotes the Moore-Penrose pseudo-inverse. In turn, the residual part, $\mathbf{\Omega}_\perp$, can be written as $\mathbf{\Omega}_\perp = (\mathbf{I} - \mathbf{F}\mathbf{F}^+)\mathbf{\Omega}$. Accordingly,

$$\begin{aligned}\mathbf{\Omega} &= \mathbf{F}\mathbf{F}^+\mathbf{\Omega} + (\mathbf{I} - \mathbf{F}\mathbf{F}^+)\mathbf{\Omega}, \\ &\equiv \mathbf{\Omega}_F + \mathbf{\Omega}_\perp.\end{aligned}$$

In order to show that the recurrent weights $\mathbf{\Omega}$ converge to $\mathbf{\Omega}_F$, we will prove that the learning rule eliminates the residual part, $\mathbf{\Omega}_\perp$. The update of $\mathbf{\Omega}_\perp$ is the corresponding projection of the total update of the recurrent weights (S.28),

$$\begin{aligned}\Delta\mathbf{\Omega}_\perp &= (\mathbf{I} - \mathbf{F}\mathbf{F}^+)\Delta\mathbf{\Omega}, \\ &= (\mathbf{I} - \mathbf{F}\mathbf{F}^+)\left(-2\mathbf{F}\mathbf{x}\mathbf{e}_{k(j)}^\top - \mathbf{\Omega}\left(2\mathbf{r} + \mathbf{e}_{k(j)}\right)\mathbf{e}_{k(j)}^\top\right), \\ &= -\mathbf{\Omega}_\perp\left(2\mathbf{r} + \mathbf{e}_{k(j)}\right)\mathbf{e}_{k(j)}^\top. \end{aligned} \tag{S.31}$$

where the last step follows from the relation $\mathbf{F}\mathbf{F}^+\mathbf{F} = \mathbf{F}$. In order to confirm convergence, we need to prove that the mean update $\langle\Delta\mathbf{\Omega}_\perp\rangle_{\text{spikes}}$ always decreases the norm of $\|\mathbf{\Omega}_\perp\|^2$, i.e. we need to show that

$$\begin{aligned}\|\mathbf{\Omega}_\perp\|^2 &\geq \left\|\mathbf{\Omega}_\perp + \epsilon\left\langle\Delta\mathbf{\Omega}_\perp\right\rangle_{\text{spikes}}\right\|^2, \\ &= \|\mathbf{\Omega}_\perp\|^2 + 2\epsilon\,\mathrm{tr}\left[\mathbf{\Omega}_\perp^\top\left\langle\Delta\mathbf{\Omega}_\perp\right\rangle_{\text{spikes}}\right] + \mathcal{O}(\epsilon^2),\end{aligned}$$

which results in the inequality

$$0 \geq \mathrm{tr}\left[\mathbf{\Omega}_\perp^\top\left\langle\Delta\mathbf{\Omega}_\perp\right\rangle_{\text{spikes}}\right]. \tag{S.32}$$

Plugging in (S.31) we find

$$\begin{aligned}\mathrm{tr}\left[\mathbf{\Omega}_\perp^\top\left\langle\Delta\mathbf{\Omega}_\perp\right\rangle_{\text{spikes}}\right] &= -\,\mathrm{tr}\left[\mathbf{\Omega}_\perp^\top\left\langle\mathbf{\Omega}_\perp\left(2\mathbf{r} + \mathbf{e}_{k(j)}\right)\mathbf{e}_{k(j)}^\top\right\rangle_{\text{spikes}}\right], \\ &= -\,\mathrm{tr}\left[\mathbf{\Omega}_\perp\left\langle\left(2\mathbf{r} + \mathbf{e}_{k(j)}\right)\mathbf{e}_{k(j)}^\top\right\rangle_{\text{spikes}}\mathbf{\Omega}_\perp^\top\right], \\ &\approx -\,\mathrm{tr}\left[\mathbf{\Omega}_\perp\left\langle 2\mathbf{r}\mathbf{r}^\top/\left|\mathbf{r}\right| + \mathbf{e}_{k(j)}\mathbf{e}_{k(j)}^\top\right\rangle_{\text{spikes}}\mathbf{\Omega}_\perp^\top\right], \end{aligned} \tag{S.33}$$

where we used that for a given stimulus the rates are (to first order) fairly constant[12] over time and the average of the spike counts will be equivalent to

[12]Strictly speaking, this assumption is only valid in the limit of high instantaneous firing rates. In the regime of low firing rates, however, the (positive) diagonals in the inner bracket of (S.33) dominate and so the expectation value is still likely to be semi-positive definite as required to prove relation (S.32)

the rates, so $\left\langle \mathbf{r}\mathbf{e}_{k(j)}^\top \right\rangle_{\text{spikes}} \approx \left\langle \mathbf{r}\mathbf{r}^\top / \left|\mathbf{r}\right| \right\rangle_{\text{spikes}}$. Finally, observe that the inner bracket of (S.33) is semi-positive definite and so we proved the desired relation $\operatorname{tr}\left[\boldsymbol{\Omega}_\perp^\top \left\langle \Delta\boldsymbol{\Omega}_\perp \right\rangle_{\text{spikes}}\right] \leq 0$. Consequently, any stable network will converge to a low-rank factorization under mild assumptions.

9.4 Learning with L2 costs

As explained in section 8.1, we need to introduce quadratic costs before considering the learning of the feedforward weights. The quadratic (L2) costs change the target connectivity to $\boldsymbol{\Omega} \to -\mathbf{F}\mathbf{D} - \mu\mathbf{I}$. This target can be obtained through the following learning rule, modified from (S.26),

$$\Delta\boldsymbol{\Omega} \propto -2\big(\mathbf{V} + \mu\mathbf{r}\big)\mathbf{e}_{k(j)}^\top - (\boldsymbol{\Omega} + \mu\mathbf{I})\mathbf{e}_{k(j)}\mathbf{e}_{k(j)}^\top,$$

or, without the burden of seeing through the matrix-vector notation,

$$\Delta\Omega_{nk} \propto \begin{cases} -2\left(V_n + \mu r_n\right) - \Omega_{nk} - \mu\delta_{nk} & \text{if } k \text{ spiked,} \\ 0 & \text{otherwise.} \end{cases}$$

We remind the reader that quantities such as the voltage or the instantaneous rate are here assumed to be evaluated directly *before* a spike of neuron k, i.e., $V_n = V_n^{\text{before}}(t_k)$ and $r_n = r_n^{\text{before}}(t_k)$. To show the fixed points of this modified learning rule, we follow the exact same analysis as in section 9.2, see (S.28),

$$\begin{aligned} \Delta\boldsymbol{\Omega} &\propto -2(\mathbf{V} + \mu\mathbf{r})\mathbf{e}_{k(j)}^\top - (\boldsymbol{\Omega} + \mu\mathbf{I})\mathbf{e}_{k(j)}\mathbf{e}_{k(j)}^\top \\ &\overset{\text{(S.8)}}{=} -2(\mathbf{F}\mathbf{x} + (\boldsymbol{\Omega} + \mu\mathbf{I})\mathbf{r})\mathbf{e}_{k(j)}^\top - (\boldsymbol{\Omega} + \mu\mathbf{I})\mathbf{e}_{k(j)}\mathbf{e}_{k(j)}^\top \\ &= -2\mathbf{F}\mathbf{x}\mathbf{e}_{k(j)}^\top - (\boldsymbol{\Omega} + \mu\mathbf{I})\left(2\mathbf{r} + \mathbf{e}_{k(j)}\right)\mathbf{e}_{k(j)}^\top. \end{aligned}$$

Compared to (S.28) we only replaced $\boldsymbol{\Omega}$ by $\boldsymbol{\Omega} + \mu\mathbf{I}$. Consequently, all arguments concerning the fixed points $-\mathbf{F}\mathbf{D}$ and convergence in section 9.2 and 9.3 now hold for $\boldsymbol{\Omega} + \mu\mathbf{I}$, and so we proved $\boldsymbol{\Omega} \to -\mathbf{F}\mathbf{D} - \mu\mathbf{I}$. Following the same argument, the fixed points (S.30) of the decoder do not change.

10 Feedforward weights: Voltage-based learning

In this section, we will explain the voltage-based learning rules for the feedforward weights. (In the main text, the core intuitions are graphically explained in Figure 3.) We will first focus on conditions (3) and (4) explained in Section 6, just as with the current-based rules. We will then extend learning to include non-whitened inputs and L1 costs. The latter two generalizations achieve the full flexibility in learning promised initially.

10.1 Condition 3: Feedforward weights mimic decoder

In order to solve the full quadratic optimization problem (S.4) we need to ensure that the feedforward weights $\mathbf{F}$ align with the decoder $\mathbf{D}^\top$. To this end, we remind the reader that the decoder will converge to (S.30),

$$\mathbf{D}_n = 2\left\langle \mathbf{x} - \hat{\mathbf{x}} \right\rangle_{\text{n spikes}} .$$

In principle we would like to use this quantity to guide learning of the feedforward weights $\mathbf{F}$, just as we did in section 8.2. From a biophysical point of view, however, we cannot assume that the feedforward weights have access to the error $\mathbf{x} - \hat{\mathbf{x}}$ (only to projections of the error). Fortunately the difference between $\mathbf{x} - \hat{\mathbf{x}}$ will be proportional to the input signal $\mathbf{x}$, on average, since we assumed that the quadratic costs are non-negligible (see previous section). To see why that is the case, we note that, once the recurrent connections have been learnt, the voltages of the neurons are in the balanced state, i.e., their averages are zero. We can therefore write (on average):

$$\langle \mathbf{V} \rangle = \langle \mathbf{F}(\mathbf{x} - \hat{\mathbf{x}}) - \mu \mathbf{r} \rangle \approx 0$$

Multiplying be the decoder from the left and re-arranging, we obtain

$$\begin{aligned} \big\langle \mathbf{DF}(\mathbf{x} - \hat{\mathbf{x}}) - \mu \hat{\mathbf{x}} \big\rangle &\approx 0 \\ \big\langle (\mathbf{DF} + \mu \mathbf{I})(\mathbf{x} - \hat{\mathbf{x}}) \big\rangle &\approx \mu \mathbf{x} \\ \big\langle (\mathbf{x} - \hat{\mathbf{x}}) \big\rangle &\approx \mu (\mathbf{DF} + \mu \mathbf{I})^{-1} \mathbf{x} \end{aligned}$$

For sufficiently large μ, the errors are therefore proportional to $\mathbf{x}$. Furthermore, as shown in the next section, when the feedforward weights converge to the transpose of the decoder, then $\mathbf{D}$ becomes an orthogonal matrix so that $\mathbf{DD}^\top \propto \mathbf{I}$, and the relation holds even if μ is small.

These costs will prohibit $\hat{\mathbf{x}}$ to fully match the size of $\mathbf{x}$, an effect that increases linearly with the size of $\mathbf{x}$. Accordingly, input signal and error are, on average, proportional to each other, i.e., $\mathbf{x} - \hat{\mathbf{x}} \propto \mathbf{x}$. The learning rule for the feedforward connections can therefore be approximated by:

$$\Delta \mathbf{F}_n(t_j) \propto \begin{cases} \mathbf{x}(t_j) - \mathbf{F}_n & \text{if } n \text{ spiked,} \\ 0 & \text{otherwise.} \end{cases} \tag{S.34}$$

Importantly, we note that, if the current $\mathbf{c}$ is changing slowly compared to the dynamics of the network, any sufficiently leaky integration of $\mathbf{c}$ is a good

approximation of $\mathbf{x} - \hat{\mathbf{x}}$. This observation becomes particularly important in the case of faster inputs: here the error $\mathbf{x} - \hat{\mathbf{x}}$ can become dominated by the inability of the network to follow the inputs, so that a stable equilibrium is never reached. In such cases we often find numerically that a less leaky integration of the current leads to more efficient networks and better reconstruction errors. For the sake of mathematical precision, we will here make the assumption that $\mathbf{c}$ is changing slowly, as also stated at the very beginning, section 1.1, and we will proceed with (S.34).

10.2 Condition 4: Feedforward weights reach optimum

Analysing the stable fixed points from the interacting feedforward and recurrent synaptic plasticity rules is daunting since the membrane voltage of each cell depends on the exact sequence and timing of the spikes. Under these conditions there is little we can do beyond the numerical simulations (see main text). For large networks, however, even small noise sources will considerably randomize the timings and sequences of spikes [1], and so in this limit it makes sense to analyse the fixed points under the assumption that spikes are distributed according to an inhomogeneous Poisson process with mean firing rates $\bar{r}_k(\mathbf{x})$. In this case the fixed point of the feedforward learning rule, (S.34), is simply given by computing the expectation value over stimuli,

$$\mathbf{F}^* = \left\langle \bar{\mathbf{r}}\mathbf{x}^\top \right\rangle .$$

Since the feedforward weights align with the decoder by design, the latter has the same fixed point (up to a transpose), so that

$$\mathbf{D}^* = \left\langle \mathbf{x}\bar{\mathbf{r}}^\top \right\rangle .$$

By multiplying with $\mathbf{D}^{*\top}\mathbf{D}^*$ from the right we can identify a simple condition on the fixed point,

$$\begin{aligned} \mathbf{D}^*\mathbf{D}^{*\top}\mathbf{D}^* &= \left\langle \mathbf{x}\bar{\mathbf{r}}^\top \right\rangle \mathbf{D}^{*\top}\mathbf{D}^* \\ &= \left\langle \mathbf{x}(\mathbf{D}^*\bar{\mathbf{r}})^\top \right\rangle \mathbf{D}^* \\ &= \left\langle \mathbf{x}\hat{\mathbf{x}}^\top \right\rangle \mathbf{D}^*. \end{aligned}$$

As observed above, the reconstruction $\hat{\mathbf{x}}$ will closely follow the input signal $\mathbf{x}$, only slightly scaled down due to the quadratic costs. Since the input signal was assumed to be white, $\langle \mathbf{x}\mathbf{x}^\top \rangle = \mathbf{I}$, we can conclude that $\langle \mathbf{x}\hat{\mathbf{x}}^\top \rangle \lessapprox \mathbf{I}$. Hence,

$$\mathbf{D}^*\mathbf{D}^{*\top}\mathbf{D}^* \lessapprox \mathbf{D}^*.$$

This condition is only fulfilled if the transpose of $\mathbf{D}^*$ is its own pseudo-inverse, and so $\mathbf{D}^*$ is a unitary matrix. (Or, more precisely, a slightly scaled down version of a unitary matrix.) In other words, in its fixed points the network represents the independent axes of a white stimulus on orthogonal directions of the population response $\mathbf{r}$, which is optimal. We discuss the non-whitened inputs in the section 12.

11 Learning with L1 costs

In the section 9.4, we have already explained how an L2 cost term modifies the learning rules for the recurrent weights. In this section, we discuss an L1 cost term, which affects both feedforward and recurrent weights.

11.1 Scaling of the synaptic weights

So far, we have ignored the relationship between the threshold T_n and the scale of the final decoder, see Equation (S.15). As explained there, we interpret the threshold as a length-constraint on the decoder. This length-constraint effects the scaling of both the feedforward and recurrent weights. Using our knowledge of the optimal architecture, $\mathbf{F}^\top = \mathbf{D}$ and $\boldsymbol{\Omega} = -\mathbf{D}^\top\mathbf{D} - \mu\mathbf{I}$, we can directly interpret the constraint on the decoder as constraints on the feedforward and recurrent connectivity, namely

$$\|\mathbf{F}_n\|_2^2 = 2T_n - \mu - \nu, \tag{S.35}$$

$$\Omega_{nn} = -\|\mathbf{D}_n\|_2^2 - \mu = -2T_n + \nu. \tag{S.36}$$

These scaling constraints need to be taken into account when learning the synaptic connectivity. However, it is important to note that the constraints only change the scale but not the structure of the network connectivity.

Whereas the L2 cost modifies the recurrent connectivity, so that $\boldsymbol{\Omega} \to -\mathbf{D}^\top\mathbf{D} - \mu\mathbf{I}$, the L1 cost *only* enters the learning through these two equations. Since we assume that the thresholds of the neurons are given some initial value and are then never changed, our learning rules need to be modified in order to account for the appropriate scale of the synaptic weights.

11.2 Modified learning of recurrent weights

To guarantee the relation $\Omega_{nn} = -2T_n + \nu$, we introduce a scaling factor β_n for every neuron n in the learning rule of the recurrent synapses,

$$\Delta\Omega_{nk} \propto \begin{cases} -\beta_n(V_n + \mu r_n) - \Omega_{nk} - \mu\delta_{nk} & \text{if } k \text{ spiked,} \\ 0 & \text{otherwise.} \end{cases} \tag{S.37}$$

Following again section 9.2 and the appropriate correction for the L2 costs in section 9.4, it is straight-forward to see that $\boldsymbol{\Omega}$ will converge to $-\mathbf{FD} - \mu\mathbf{I}$, where the decoder $\mathbf{D}$ is now modified by the scaling factor β_n so that

$$D_{in} \to \beta_n \langle x_i - \hat{x}_i \rangle_{\text{n spikes}} .$$

Hence, in order to obey (S.36), the scaling factors β_n should evolve according to,

$$\begin{aligned} -\Omega_{nn} &= 2T_n - \nu \\ \Leftrightarrow \mathbf{F}_n^\top \mathbf{D}_n + \mu &= 2T_n - \nu, \\ \Leftrightarrow \mathbf{F}_n^\top \beta_n \langle \mathbf{x} - \hat{\mathbf{x}} \rangle_{\text{n spikes}} + \mu &= 2T_n - \nu, \\ \Leftrightarrow \beta_n &= \frac{2T_n - \mu - \nu}{\mathbf{F}_n^\top \langle \mathbf{x} - \hat{\mathbf{x}} \rangle_{\text{n spikes}}}, \\ \Leftrightarrow \beta_n &= \frac{2T_n - \mu - \nu}{T_n + \mu \langle r_n \rangle_{\text{n spikes}}} \end{aligned} \tag{S.38}$$

where in the last step we have used the relation $\mathbf{F}_n^\top \langle \mathbf{x}-\hat{\mathbf{x}} \rangle_{\text{n spikes}} = \langle V_n \rangle_{\text{n spikes}} + \mu \langle r_n \rangle_{\text{n spikes}}$ and $V_n = V_n^{\text{before}} = T_n$, since the voltage directly before the spike of the firing neuron is, by definition, the neuron's threshold. Note that in the absence of costs, $\mu = \nu = 0$, we recover $\beta_n = 2$, i.e., the unscaled learning rule (S.24).

11.3 Modified learning for feedforward weights

Similarly, to guarantee the scaling $\|\mathbf{F}_n\|_2^2 = 2T_n - \mu - \nu$ of the feedforward weights, we introduce appropriate scaling factors α_n into the learning rule (S.34),

$$\Delta \mathbf{F}_n \propto \begin{cases} \alpha_n \mathbf{x} - \mathbf{F}_n & \text{if } n \text{ spiked,} \\ 0 & \text{otherwise.} \end{cases} \tag{S.39}$$

which will consequently lead to a scaling of the fixed point,

$$\mathbf{F}_n \to \alpha_n \langle \mathbf{x} \rangle_{\text{n spikes}} .$$

Hence, in order to fulfill (S.35) it should hold that

$$\begin{aligned} \|\mathbf{F}_n\|^2 &= \mathbf{F}_n^\top \mathbf{F}_n \\ &= \mathbf{F}_n^\top \alpha_n \langle \mathbf{x} \rangle_{\text{n spikes}} \\ &= 2T_n - \mu - \nu \end{aligned}$$

from which we read off an expression for the scaling factors,

$$\alpha_n = \frac{2T_n - \mu - \nu}{\mathbf{F}_n^\top \langle \mathbf{x} \rangle_{\text{n spikes}}}. \tag{S.40}$$

The learning rules (S.37) and (S.39) in conjunction with the definition of the scaling factors (S.38) and (S.40) are thus the set of rules that take into account all costs and will make the network converge to the optimal network configuration with the optimal decoding weights.

11.4 Simplifying assumptions and final learning rules

The scaling factors α_n and β_n for the feedforward and recurrent weights guarantee the convergence of the network to the properly scaled weights. It is important to remember that the scaling factors only set the right scale of the weights, they do not affect the overall structure of the optimal connectivities, $\mathbf{F} \propto \mathbf{D}^\top$ and $\mathbf{\Omega} \propto -\mathbf{D}^\top \mathbf{D} - \mu \mathbf{I}$. In addition, we note that fixing the scaling factors α_n and β_n merely fixes a set of fixed points with a particular L1 cost ν and scaling of $\mathbf{D}$.

To simplify these learning rules, one could therefore also simply fix the scaling factors, e.g. by setting $\alpha_n = \alpha$ and $\beta_n = \beta$ (which assumes that all thresholds have the same values $T_n = T$). All weights will then converge to the right connectivity structure, but the effective L1 cost, or the specific choice of ν, remains a priori unknown.

Strictly speaking, α_n and β_n are related to each other (compare eqs. S.40 and S.38). In practice, however, we simply set α and β by hand such that

the fixed points exhibit reasonable cost values and scales. This approximation worked quite well. The recurrent learning rule, (S.37), then becomes

$$\Delta\Omega_{nk} \propto \begin{cases} -\beta(V_n + \mu r_n) - \Omega_{nk} - \mu\delta_{nk} & \text{if } k \text{ spiked,} \\ 0 & \text{otherwise.} \end{cases} \tag{S.41}$$

which, when rewritten as a differential equation, is equivalent to Equation (6) shown in the main paper. The feedforward rule, (S.39), becomes

$$\Delta F_{ni} \propto \begin{cases} \alpha x_i - F_{ni} & \text{if } n \text{ spiked,} \\ 0 & \text{otherwise.} \end{cases} \tag{S.42}$$

When rewritten as a differential equation, this rule is equivalent to Equation (7) in the main paper. In both rules, α and β are simply treated as free parameters.

12 Learning rules for non-whitened inputs

So far we have assumed that the input stimulus is zero-mean and whitened. To cover more general scenarios, we first revisit the optimal spiking neural network from section 4, following the approach outlined in [6] for rate networks. First, we note that a self-organized network is incapable of determining the true covariance of the signal (which could always be "arbitrarily" distorted by the feedforward weights) while the mean of the signal should be filtered out to increase efficiency (otherwise spikes are constantly emitted just to support a fixed offset). To take both aspects into account, we modify the loss function (S.6),

$$\ell = (\mathbf{x}_c - \mathbf{D}\mathbf{r})^\top \mathbf{C}^{-1} (\mathbf{x}_c - \mathbf{D}\mathbf{r}) + \mu \|\mathbf{r}\|^2 + \nu \|\mathbf{r}\|_1 \tag{S.43}$$

where $\mathbf{x}_c = \mathbf{x} - \bar{\mathbf{x}}$ is the mean signal and $\mathbf{C} = \langle \mathbf{x}_c \mathbf{x}_c^\top \rangle$ is the signal covariance. It is important to note that the more general loss (S.43) is invariant with respect to linear transformations in the input $\mathbf{x}$. If the input signal is replaced by a transformed signal $\mathbf{x}' := \mathbf{M}\mathbf{x}$, with $\mathbf{M}$ an invertible matrix, the loss function is not affected. It is sufficent to replace $\mathbf{D}$ with $\mathbf{MD}$ to recover an equivalent optimization problem. In complete analogy to section 4, one can derive the voltages and thresholds of simple integrate-and-fire neurons,

$$\mathbf{V} = \mathbf{D}^\top \mathbf{C}^{-1} \mathbf{x}_c - \mathbf{D}^\top \mathbf{C}^{-1} \mathbf{D}\mathbf{r} - \mu \mathbf{r},$$
$$T_n = \frac{1}{2} \left(\|\mathbf{D}_n\|^2 + \mu + \nu \right).$$

Accordingly, the network is now characterized by feedforward weights $\mathbf{F} = \mathbf{D}^\top \mathbf{C}^{-1}$ and recurrent weights $\mathbf{\Omega} = -\mathbf{D}^\top \mathbf{C}^{-1}\mathbf{D} - \mu \mathbf{I} = -\mathbf{F}\mathbf{D} - \mu \mathbf{I}$. These equations show that we only need to revisit the feedforward weights, whose relation to the decoder has changed, but not the recurrent weights, whose relation to the feedforward and decoder weights remains the same. Indeed, we did not make any (implicit or explicit) assumptions on the statistics of the input in the derivation of the recurrent learning rules, and so the same learning rule (S.37) applies in this case.

To make the feedforward weights converge to $\mathbf{F} = \mathbf{D}^\top \mathbf{C}^{-1}$, we modify the learning rule (S.34) as

$$\Delta \mathbf{F}_n \propto \begin{cases} \mathbf{x}_c - \mathbf{F}_n^\top \mathbf{x}_c \mathbf{x}_c & \text{when neuron } n \text{ spikes,} \\ 0 & \text{otherwise.} \end{cases} \tag{S.44}$$

We emphasize that this learning rule is still local. We can highlight this feature by stating the learning rule for the i-th element of $\mathbf{F}_n$,

$$\Delta F_{in} \propto \begin{cases} [x_c]_i - (\mathbf{F}_n^\top \mathbf{x}_c)[x_c]_i & \text{when neuron } n \text{ spikes,} \\ 0 & \text{otherwise.} \end{cases}$$

Here, $\mathbf{F}_n^\top \mathbf{x}_c$ is simply the total feedforward current that the postsynaptic neuron received. Accordingly, the modified learning rule requires a multiplicative, yet local interaction between the presynaptic signal, $[x_c]_i$, and the postsynaptic current. In Figure 3 of the main text, we illustrate this modified learning rule in a network of six neurons that receive a correlated input signal. Interestingly, the network learns tuning curves that are narrower and denser around the most

frequently presented signal directions. This is reminiscent of the tuning curves derived from efficient coding principles in population of Poisson-firing neurons [20].

Following the derivation of section 10.2, and assuming once more Poisson-distributed spike trains, the fixed points, $\mathbf{F}^*$, of the feedforward rule become

$$\begin{aligned}\left\langle \bar{\mathbf{r}}\mathbf{x}_c^\top - \mathbf{F}^*\mathbf{x}_c\mathbf{x}_c^\top \right\rangle &= 0,\\ \Leftrightarrow \mathbf{F}^* \left\langle \mathbf{x}_c\mathbf{x}_c^\top \right\rangle &= \left\langle \bar{\mathbf{r}}\mathbf{x}_c^\top \right\rangle,\\ \Rightarrow \mathbf{F}^* &= \mathbf{D}^{*\top}\mathbf{C}^{-1}.\end{aligned}$$

where the fixed point of the decoder, $\mathbf{D}^*$ remains untouched, and becomes

$$\mathbf{D}^* \propto \left\langle \mathbf{x}_c\bar{\mathbf{r}}^\top \right\rangle.$$

Using this relation once more, and multiplying it with $\mathbf{D}^{*\top}\mathbf{C}^{-1}\mathbf{D}^*$ from the right, we find the following relation for the decoder

$$\begin{aligned}\mathbf{D}^*\mathbf{D}^{*\top}\mathbf{C}^{-1}\mathbf{D}^* &\propto \left\langle \mathbf{x}_c\bar{\mathbf{r}}^\top \right\rangle \mathbf{D}^{*\top}\mathbf{C}^{-1}\mathbf{D}^*,\\ &= \left\langle \mathbf{x}_c(\mathbf{D}^*\bar{\mathbf{r}})^\top \right\rangle \mathbf{C}^{-1}\mathbf{D}^*,\\ &= \left\langle \mathbf{x}_c\hat{\mathbf{x}}_c \right\rangle \mathbf{C}^{-1}\mathbf{D}^*,\\ &\underset{\sim}{\propto} \mathbf{D}^*,\end{aligned}$$

which is fulfilled if $\mathbf{D}^* = \mathbf{C}^{1/2}\mathbf{U}$, where $\mathbf{U}$ is a unitary matrix. Then $\mathbf{F}^* = \mathbf{D}^{*\top}\mathbf{C}^{-1} = \mathbf{U}^\top\mathbf{C}^{-1/2}$. This last equation exposes the solution that the network finds: it whitens the input through its feedforward filters before encoding it along orthogonal axes in the population response.

13 Learning in the EI network

So far we neglected Dale's law, i.e., the distinction between excitatory and inhibitory neurons. We will first introduce the full equations for the EI network, and then describe how the learning rules can be applied to this network.

13.1 Voltage dynamics

We assume that the membrane voltages of both the excitatory and inhibitory neurons follow the dynamics of current-based, leaky integrate-and-fire neurons. Specifically, the voltage V_n^E of the n-th excitatory neuron is given by

$$\dot{V}_n^E(t) \equiv \frac{\partial V_n^E}{\partial t} = -\lambda V_n^E(t) + \mathbf{F}_n^E \cdot \mathbf{c}(t) + \boldsymbol{\Omega}_n^{EE} \cdot \mathbf{o}^E(t) + \boldsymbol{\Omega}_n^{EI} \cdot \mathbf{o}^I(t) + \sigma\eta(t), \quad \text{(S.45)}$$

where $\mathbf{F}_n^E$ are the feedforward weights of neuron n, $\boldsymbol{\Omega}_n^{EE}$ are the weights of the recurrent excitatory inputs, $\boldsymbol{\Omega}_n^{EI}$ are the weights of the recurrent inhibitory inputs, and $\sigma\eta(t)$ is a noise term. The multiplication sign '$\cdot$' denotes the inner product or dot product. We generally assume that the feedforward weights can be either excitatory or inhibitory, meaning that individual elements of $\mathbf{F}_n^E$ can be either positive or negative. The elements of $\boldsymbol{\Omega}_n^{EI}$ are assumed to be negative and the elements of $\boldsymbol{\Omega}_n^{EE}$ are assumed to be positive, with one exception: the self-connection weight Ω_{nn}^{EE} is assumed to be negative, as it determines the neuron's reset potential after a spike. Whenever the neuron hits a threshold, T_n^E, it fires a spike and resets its own voltage. In other words, we have included the reset of the integrate-and-fire neuron in its self-connection for mathematical convenience. After each spike, the voltage is therefore reset to $V_n^E \to T_n^E + \Omega_{nn}^{EE}$, where Ω_{nn}^{EE} is a negative number.

Similarly, the membrane voltage V_n^I of the n-th inhibitory neuron follows the dynamics

$$\dot{V}_n^I(t) \equiv \frac{\partial V_n^I}{\partial t} = -\lambda V_n^I(t) + \boldsymbol{\Omega}_n^{IE} \cdot \mathbf{o}^E(t) + \boldsymbol{\Omega}_n^{II} \cdot \mathbf{o}^I(t) + \sigma\eta(t), \quad \text{(S.46)}$$

where $\boldsymbol{\Omega}_n^{IE}$ are the recurrent weights from the excitatory population and $\boldsymbol{\Omega}_n^{II}$ are the recurrent weights from the inhibitory population. The thresholds are given by T_n^I and the reset is contained in the element Ω_{nn}^{II} of the recurrent inhibitory input.

13.2 Learning the synapses

Using the intuition from the development of the learning rules in the non-Dalian network, it is straightforward to see how learning in an EI network should proceed. Consider a population of excitatory neurons that receives feedforward input. E-E connections are constrained to be excitatory, and so neurons with overlapping inputs cannot balance each other. If, however, a population of inhibitory neurons has learned to represent the signal encoded by the excitatory population, then its representation can in turn be used to balance the excitatory population. This suggests that the E-I connections are to be treated like feedforward connections because the excitatory population response serves as the input to the inhibitory population. The E-E and I-I connections are to be

trained by the recurrent learning rule since they aim to balance the E- and the I-population respectively. Finally, the I-E connections should be trained the same way since they aim to balance the E-population.

We can formalize this intuition as follows. First, consider the optimal, non-Dalian network without costs, $\mathbf{\Omega} = -\mathbf{D}\mathbf{D}^\top$, and split the decoder weights $\mathbf{D} = \mathbf{D}_+ - \mathbf{D}_-$ into one part with all positive and another with the absolute value of all negative entries. Then,

$$\begin{aligned}\mathbf{\Omega}\mathbf{r} &= -(\mathbf{D}_+ - \mathbf{D}_-)^\top(\mathbf{D}_+ - \mathbf{D}_-)\mathbf{r}, \\ &= (\mathbf{D}_-^\top\mathbf{D}_+ + \mathbf{D}_+^\top\mathbf{D}_-)\mathbf{r} - (\mathbf{D}_+^\top\mathbf{D}_+ + \mathbf{D}_-^\top\mathbf{D}_-)\mathbf{r}.\end{aligned}$$

We can identify the first term as the recurrent excitation and the second term as the recurrent inhibition. It is this latter term that we need to approximate by means of an inhibitiory population. To this end let $\mathbf{r}$ be approximated by the response $\mathbf{s}$ of a second population, i.e. $\hat{\mathbf{r}} = \tilde{\mathbf{D}}\mathbf{s}$ and so

$$\mathbf{\Omega}\mathbf{r} \approx (\mathbf{D}_-^\top\mathbf{D}_+ + \mathbf{D}_+^\top\mathbf{D}_-)\mathbf{r} - (\mathbf{D}_+^\top\mathbf{D}_+ + \mathbf{D}_-^\top\mathbf{D}_-)\tilde{\mathbf{D}}\mathbf{s}.$$

The second population shall minimize the objective

$$L_I = \arg\min_{\tilde{\mathbf{D}}} \left\langle \left\| \mathbf{r} - \tilde{\mathbf{D}}\mathbf{s} \right\|^2 \right\rangle,$$

which we know is solved optimally by a network with feedforward weights $\tilde{\mathbf{D}}^\top$ and recurrent weights $-\tilde{\mathbf{D}}\tilde{\mathbf{D}}^\top$. Observe that this second population has only inhibitory recurrent weights and its influence on the first is solely inhibitory; it can therefore be identified as an inhibitory population. At the same time, the first population has only excitatory recurrent weights and its influence on the second is solely excitatory; it can therefore be identified as an excitatory population.

In summary, the structure of the optimal EI network is given by feedforward weights $\mathbf{D}^\top$, E-E connections $\mathbf{\Omega}_{EE} = \mathbf{D}_-^\top\mathbf{D}_+ + \mathbf{D}_+^\top\mathbf{D}_-$, E-I connections $\mathbf{\Omega}_{IE} = \tilde{\mathbf{D}}^\top$, I-I connections $\mathbf{\Omega}_{II} = -\tilde{\mathbf{D}}\tilde{\mathbf{D}}^\top$ and I-E connections $\mathbf{\Omega}_{EI} = -(\mathbf{D}_+^\top\mathbf{D}_+ + \mathbf{D}_-^\top\mathbf{D}_-)\tilde{\mathbf{D}}$. From the derivation it is clear that $\mathbf{\Omega}_{IE}$ act as feedforward weights to the inhibitory population and are thus to be trained by the standard feedforward rule. All other weights, i.e. $\mathbf{\Omega}_{EE}, \mathbf{\Omega}_{IE}$ and $\mathbf{\Omega}_{EI}$, are trained using the recurrent learning rule. The training then proceeds as in the non-Dale's case except for the sign constraint on the synaptic weights.

14 Numerical Simulations

In this section, we detail how we simulated the learning rules in practice, and we provide pseudo-code for all simulations. The MATLAB code for the figures of the main paper will be made available on http://www.github.com/machenslab/spikes.

14.1 Network Dynamics

The membrane voltage V_n of each cell is simulated according to a discrete-time (Euler) approximation of the differential equations, either (S.45) and (S.46) for the EI networks, or (S.7) for the non-Dalian networks,

$$V_n(t + \Delta t) = V_n(t) + \Delta t \dot{V}_n(t).$$

In Figure 2 and 4, the target signal $\mathbf{x}(t)$ is set as follows: we first draw a random vector $\mathbf{y}(t) \in \mathbf{R}^M$ from a zero-mean Gaussian distribution $\mathbf{y}(t) \sim \mathcal{N}(0, \sigma^2 \mathbf{I})$ for every time-step t and then convolve $\mathbf{y}(t)$ with a Gaussian kernel of size η over time to get $\mathbf{x}(t)$. For Figure 6, the target signal was the speech spectrogram, sampled at 100Hz and interpolated to reach a temporal resolution of 0.05 ms. The input current $\mathbf{c}(t)$ is computed following (S.1), i.e.,

$$c_i(t) = \dot{x}_i(t) + \lambda x_i(t).$$

Furthermore, gaussian noise terms ξ_V and ξ_T with very small variances and zero means are added respectively to the voltage equation and to the thresholds of the neurons.

14.2 Learning

In all simulations, the recurrent (and I to E) weights are trained by means of the recurrent learning rule (S.41).

The feedforward weights $\mathbf{F}$ and the $E-I$ connection follows (S.42) (whitened inputs, Figure 2, 3A,B, and 4) or (S.44) (non-whitened input, Figure 3C,D, 6,7). However, in Figure 4–8, the input signal $\mathbf{x}$ in equations (S.42) and (S.44) is replaced by the input currents integrated with a larger leak term $\lambda_F > \lambda$. For example, in Figure 4, the learning rule of the feedforward connection is

$$\Delta \mathbf{F}_n(t_j) \propto \begin{cases} \bar{\mathbf{c}}(t_j) - \mathbf{F}_n & \text{if } n \text{ spiked,} \\ 0 & \text{otherwise.} \end{cases}$$

where $\bar{\mathbf{c}}$ obeys $\dot{\bar{\mathbf{c}}} = -\lambda_F \bar{\mathbf{c}} + \mathbf{c}$.

The constant scaling term α is chosen so as to achieve mean firing rates of around 5 to 10 Hz after training. The learning rates ϵ_F and ϵ_Ω of the feedforward and recurrent weights are either kept constant throughout the simulation (Figures 2–4), or progressively decreased (Figure 6). Importantly, there is a separation of time-scales such that $\epsilon_\Omega = 10\epsilon_F$; this ensures that the network is always kept in a balanced (and thus stable) regime throughout learning.

The full pseudo-code for the non-Dales case can be found in algorithm 1.

Algorithm 1 Pseudo-code for simulation of non-Dales network (Figures 2, 5–7)

procedure SIMULATION
  $N, M \leftarrow$ number of cells and input dimensions
  $\lambda \leftarrow$ membrane leak
  $\mathbf{F}(0) \leftarrow$ initial feedforward weights
  $\mathbf{\Omega}(0) \leftarrow$ initial recurrent weights
  $S \leftarrow$ total simulation time
  $\mathrm{dt} \leftarrow$ time-step
  $\epsilon_F, \epsilon_\Omega \leftarrow$ learning rates
  $\alpha, \beta \leftarrow$ scaling factors in learning equations
  $\mu \leftarrow$ L2 cost
  $T \leftarrow$ threshold
  $\sigma, \eta \leftarrow$ standard deviation of signal, time-scale of smoothing kernel
top:
  $\mathbf{V}(0) \leftarrow \mathbf{0}$ (initial voltage)
  $\mathbf{o}(0) \leftarrow \mathbf{0}$ (initial spikes)
  $\mathbf{r}(0) \leftarrow \mathbf{0}$ (initial filtered spikes)
  $\Gamma \leftarrow$ closest integer to S/dt (number of simulation steps)
  $\mathbf{x}(\tau) \leftarrow$ drawn from $\mathcal{N}(0, \sigma^2 \mathbf{I}_M)$ for all $\tau = 1 \ldots \Gamma$
  $\mathbf{x}(\tau) \leftarrow \mathbf{x}(\tau)$ filtered with Gaussian kernel of width η over time
  $\xi_V(\tau) \leftarrow$ drawn from $\mathcal{N}(0, \sigma^2_{\xi_V} \mathbf{I}_N)$ for all $\tau = 1 \ldots \Gamma$
  $\xi_T(\tau) \leftarrow$ drawn from $\mathcal{N}(0, \sigma^2_{\xi_T} \mathbf{I}_N)$ for all $\tau = 1 \ldots \Gamma$
loop:
  for $\tau = 1$ **to** Γ **do**
    $\mathbf{c}(\tau-1) = \mathbf{x}(\tau) - \mathbf{x}(\tau-1) + \lambda\,\mathrm{dt}\,\mathbf{x}(\tau-1)$
    $\mathbf{V}(\tau) = (1-\lambda\,\mathrm{dt})\mathbf{V}(\tau-1) + \mathrm{dt}\,\mathbf{F}(\tau-1)^\top \mathbf{c}(\tau-1) + \mathbf{\Omega}(\tau-1)\mathbf{o}(\tau-1) + \xi_V(\tau)$

    $\mathbf{o}(\tau) = \mathbf{0}$
    $n \leftarrow \arg\max\,(\mathbf{V} - \mathbf{T}) - \xi_T(\tau))$
    if $V_n > T_n$ **then**
      $o_n(\tau) = 1$
      $\mathbf{F}_n(\tau) = \mathbf{F}_n(\tau-1) + \epsilon_F(\alpha\mathbf{x}(\tau-1) - \mathbf{F}_n(\tau-1))$
      $\mathbf{\Omega}_n(\tau) = \mathbf{\Omega}_n(\tau-1) - \epsilon_\Omega(\beta(\mathbf{V}(\tau-1) + \mu\mathbf{r}(\tau-1)) + \mathbf{\Omega}_n(\tau-1))$
      $\Omega_{nn}(\tau) = \Omega_{nn}(\tau-1) - \epsilon_\Omega\mu$

    $\mathbf{r}(\tau) = (1-\lambda\,\mathrm{dt})\mathbf{r}(\tau-1) + \mathbf{o}(\tau-1)$

Algorithm 2 Pseudo-code for simulation of Dales network (Figure 4,8)

procedure SIMULATION
  $N_E, N_I \leftarrow$ number of cells in the excitatory and inhibitory populations
  $M \leftarrow$ number of input dimensions
  $\lambda, \lambda_F, \lambda_{EI} \leftarrow$ membrane leak and FF integration time constants
  $\mathbf{F}(0) \leftarrow$ initial feedforward weights
  $\mathbf{\Omega}^{EE}(0), \mathbf{\Omega}^{EI}(0), \mathbf{\Omega}^{II}(0), \mathbf{\Omega}^{IE}(0) \leftarrow$ initial recurrent weights
  $S \leftarrow$ total simulation time
  $\mathrm{dt} \leftarrow$ time-step
  $\epsilon_F, \epsilon_\Omega \leftarrow$ learning rates
  $\alpha, \beta \leftarrow$ scaling factors in learning equations
  $\mu \leftarrow$ L2 cost
  $T^E, T^I \leftarrow$ threshold
  $\sigma, \eta \leftarrow$ standard deviation of signal, time-scale of smoothing kernel
  $R^E, R^I \leftarrow$ refractory periods of the excitatory and inhibitory neurons
top:
  $\mathbf{V}^E(0), \mathbf{V}^I(0) \leftarrow \mathbf{0}$ (initial voltage)
  $\mathbf{o}^E(0), \mathbf{o}^I(0) \leftarrow \mathbf{0}$ (initial spikes)
  $\mathbf{r}^E(0), \mathbf{r}^I(0) \leftarrow \mathbf{0}$ (initial filtered spikes)
  $\mathbf{R}^E(0), \mathbf{R}^I(0) \leftarrow \mathbf{0}$ (initial refractory periods spikes)
  $\Gamma \leftarrow$ closest integer to S/dt (number of simulation steps)
  $\mathbf{x}(\tau) \leftarrow$ drawn from $\mathcal{N}(0, \sigma^2 \mathbf{I}_M)$ for all $\tau = 1 \dots \Gamma$
  $\mathbf{x}(\tau) \leftarrow \mathbf{x}(\tau)$ filtered with Gaussian kernel of width η over time
  $\xi_E^V(\tau), \xi_I^V(\tau) \leftarrow$ drawn from $\mathcal{N}(0, \sigma_{\xi_V}^2 \mathbf{I}_N)$ for all $\tau = 1 \dots \Gamma$
  $\xi_E^T(\tau), \xi_I^T(\tau) \leftarrow$ drawn from $\mathcal{N}(0, \sigma_{\xi_T}^2 \mathbf{I}_N)$ for all $\tau = 1 \dots \Gamma$
loop:
  for $\tau = 1$ **to** Γ **do**
    $\mathbf{c}(\tau-1) = \mathbf{x}(\tau) - \mathbf{x}(\tau-1) + \lambda\,\mathrm{dt}\,\mathbf{x}(\tau-1)$
    $\mathbf{c}_E(\tau) = (1-\lambda_F \mathrm{dt})\mathbf{c}_E(\tau-1) + \mathbf{c}(\tau-1)$
    $\mathbf{c}_I(\tau) = (1-\lambda_{EI} \mathrm{dt})\mathbf{c}_E(\tau-1) + \mathbf{o}_E(\tau-1)$
    $\mathbf{V}^E(\tau) = (1-\lambda\,\mathrm{dt})\mathbf{V}^E(\tau-1) + \mathrm{dt}\,\mathbf{F}(\tau-1)^\top \mathbf{c}(\tau-1) + \mathbf{\Omega}^{EE}(\tau-1)\mathbf{o}^E(\tau-1) + \mathbf{\Omega}^{IE}(\tau-1)\mathbf{o}^I(\tau-1) + \xi_E^V(\tau)$

    $\mathbf{o}^E(\tau) = \mathbf{0}$
    $n_E \leftarrow \arg\max\left(\mathbf{V}^E - \mathbf{T^E}\right) - \xi_E^T(\tau))$
    if $V_{n_E}^E > T_{n_E}^E \& \ R_{n_E}^E(\tau-1) < 0$ **then**
      $o_{n_E}^E(\tau) = 1$
      $R_{n_E}^E(\tau-1) = R_{max}^E$
      $\mathbf{F}_{n_E}(\tau) = \mathbf{F}_{n_E}(\tau-1) + \epsilon_F(\alpha \mathbf{c}_E(\tau-1) - \mathbf{F}_{n_E}(\tau-1))$
      $\mathbf{\Omega}_{n_E}^{EE}(\tau) = \mathbf{\Omega}_{n_E}^{EE}(\tau-1) - \epsilon_\Omega(\beta(\mathbf{V}^E(\tau-1) + \mu \mathbf{r}^E(\tau-1)) + \mathbf{\Omega}_{n_E}^{EE}(\tau-1))$
      $\Omega_{n_E n_E}^{EE}(\tau) = \Omega n_E n_E{}^{EE}(\tau-1) - \epsilon_\Omega \mu$
      $\mathbf{\Omega}_{n_E}^{EI}(\tau) = \mathbf{\Omega}_{n_E}^{EI}(\tau-1) - \epsilon_\Omega(\beta(\mathbf{V}^I(\tau-1) + \mu \mathbf{r}^I(\tau-1)) + \mathbf{\Omega}_{n_E}^{EI}(\tau-1))$

    $R^E(\tau) = R^E(\tau-1) - 1$
    $\mathbf{r}^E(\tau) = (1-\lambda\,\mathrm{dt})\mathbf{r}^E(\tau-1) + \mathbf{o}^E(\tau-1)$

$\mathbf{V}^I(\tau) = (1-\lambda\,\mathrm{dt})\mathbf{V}^I(\tau-1) + \mathbf{\Omega}^{EI}(\tau)\mathbf{o}^E(\tau) + \mathbf{\Omega}^{II}(\tau-1)\mathbf{o}^I(\tau-1) + \xi_I^V(\tau)$

$\mathbf{o}^I(\tau) = \mathbf{0}$

$n_I \leftarrow \arg\max\Big(\mathbf{V}^I - \mathbf{T^I}\Big) - \xi_I^T(\tau))$

if $V^I_{n_I} > T^I_{n_I} \,\&\, R^I_{n_I}(\tau-1) < 0$ **then**

  $o^I_{n_I}(\tau) = 1$

  $R^I_{n_I}(\tau-1) = R^I_{max}$

  $\mathbf{\Omega}^{EI}_{n_I}(\tau) = \mathbf{\Omega}^{EI}_{n_I}(\tau-1) + \epsilon_F(\alpha\mathbf{c}_I(\tau-1) - \mathbf{\Omega}^{EI}_{n_I}(\tau-1))$

  $\mathbf{\Omega}^{II}_{n_I}(\tau) = \mathbf{\Omega}^{II}_{n_I}(\tau-1) - \epsilon_\Omega(\beta(\mathbf{V}^I(\tau-1) + \mu\mathbf{r}^I(\tau-1)) + \mathbf{\Omega}^{II}_{n_I}(\tau-1))$

  $\Omega^{II}_{n_I n_I}(\tau) = \Omega n_I n_I{}^{II}(\tau-1) - \epsilon_\Omega\mu$

  $\mathbf{\Omega}^{IE}_{n_I}(\tau) = \mathbf{\Omega}^{IE}_{n_I}(\tau-1) - \epsilon_\Omega(\beta(\mathbf{V}^E(\tau-1) + \mu\mathbf{r}^E(\tau-1)) + \mathbf{\Omega}^{IE}_{n_I}(\tau-1))$

$R^I(\tau) = R^I(\tau-1) - 1$

$\mathbf{r}^I(\tau) = (1-\lambda\,\mathrm{dt})\mathbf{r}^I(\tau-1) + \mathbf{o}^I(\tau-1)$

14.3 Initialization

To initialize the feedforward weights $\mathbf{F} \in \mathbb{R}^{N\times M}$, we first draw all elements from a zero-mean normal distribution, $F_{ni} \sim \mathcal{N}(0,1)$, and then normalize each row to be of length γ, i.e.

$$F_{ni} \rightarrow \gamma \frac{F_{ni}}{\sqrt{\sum_i F_{ni}^2}}.$$

In Figures 2, and 6–8, the initial recurrent weights $\mathbf{\Omega} \in \mathbb{R}^{N\times N}$ are proportional to the unit matrix $\mathbf{I}_N$ with proportionality ω, i.e. $\mathbf{\Omega}_0 = \omega\mathbf{I}_N$. This simplified initialization is chosen for illustrative purposes; the learning also works for more general, random initializations of the recurrent connections. The recurrent connectivity in the EI network (Figure 4) is similarly initialized as $\mathbf{\Omega}_{EE} = \omega_{EE}\mathbf{I}_{N_E}$ and $\mathbf{\Omega}_{II} = \omega_{II}\mathbf{I}_{N_I}$. In all simulations there are four times more excitatory than inhibitory neurons, $N_E = 4\cdot N_I$, and so we initialize the E-I and I-E connections according to $\mathbf{\Omega}_{EI} = \omega_{EI}[\mathbf{I}_{N_I}, \mathbf{I}_{N_I}, \mathbf{I}_{N_I}, \mathbf{I}_{N_I}]$ and $\mathbf{\Omega}_{IE} = \omega_{IE}[\mathbf{I}_{N_I}, \mathbf{I}_{N_I}, \mathbf{I}_{N_I}, \mathbf{I}_{N_I}]^\top$ where the squared brackets denote a stacking of the elements along the rows. For Figures 2–4, the thresholds $T_n = T$ are kept constant over the course of the simulation (including learning) and are homogeneous across cells. For Figures 5–8, the thresholds were adjusted according to a simple homeostatic rule in order to maintain average firing rates within a pre-determined range (see also Section 14.6).

14.4 Tuning Curves

To compute the tuning curves (Figures 2–4), we define a circle in a 2D plane and then sample inputs uniformly on this circle. For each trial a constant input (orientation)is sampled from this circle and presented to the network (running without plasticity). At the end of the trial, the average firing rate of each neuron is computed over all the duration of the presentation of the input except for the initial transient period.

Parameters	Figure 2	Figure 4	Figure 6,7
Number of neurons N	20	$N_E = 300$ (4BC)<br>$N_E = 60$ (4D)<br>$N_I = 75$ (4BC)<br>$N_I = 15$ (4D)	64
Dimension of input M	2	3	25
Time step dt	10^{-3}s	10^{-4}s	$6.25 \cdot 10^{-5}$s
Membrane leak λ	50 s^{-1}	50 s^{-1}	8 s^{-1}
FF Integration time constant	$\lambda_F = \lambda$	$\lambda_F = 6\lambda$<br>$\lambda_{E-I} = \lambda$	$\lambda_F = 125\lambda$
Stdev of input σ	$2 \cdot 10^3$	$2 \cdot 10^3$	-
Time scale of input kernel η	6 ms	6 ms	-
Threshold T	0.5	0.5	dynamic
Stdev of voltage noise σ_{ξ_V}	10^{-3}	10^{-3}	0
Stdev of threshold noise σ_{ξ_T}	$2 \cdot 10^{-2}$	$2 \cdot 10^{-2}$	$5 \cdot 10^{-3}$
Learning rate ϵ_Ω	10^{-4}	10^{-4}	variable
Learning rate ϵ_F	10^{-5}	10^{-5}	variable
Scaling factor α	0.21	0.21	1
Scaling factor β	1.25	1	1
L2 cost μ	0.02	$\mu_E = 0.02, \mu_I = 0$	0.1
Initial scale γ	0.8	1	-
Initial scale ω	-0.5	$\omega_{EE} = -0.02$<br>$\omega_{II} = -0.5$<br>$\omega_{EI} = 0.5$<br>$\omega_{IE} = -0.3$	-

Table 2: Simulation parameters for all simulations.

14.5 Fano Factor and Coefficient of Variation

The Fano factor and the coefficient of variation are computed as follows. A random direction is chosen in the input space and presented multiple times to the network (running without plasticity). For each trial the spike count c of the neurons is computed. then we compute the Fano factor for each neuron using the formula :

$$F_n = \frac{\sigma^2_{c(n)}}{\mu_{c(n)}}$$

$\sigma^2_{c(n)}$ and $\mu_{c(n)}$ are respectively the standard deviation and the mean of the spike count of neuron n. This procedure is repeated using different random input directions. The final Fano factor is an average over the input directions and the neurons in the population.
The Coefficient of variation (CV) is computed using the same inputs. For each trial, instead of the spike count, we pool the interspike intervals (ISI) of all neurons. The formula used to compute the CV is

$$CV = \frac{\sigma_{ISI}}{\mu_{ISI}}$$

As for the Fano factor, the final CV is an average over the different input directions.

14.6 Simulation of speech signal learning

To learn the speech signal (Figure 5,6), slight modifications were added to the previous learning scheme. In a non-whitened scenario, partial learning of the inhibitory recurrent connections can result in a large proportion of completely silent neurons. Since plasticity require pre- and post-synaptic spiking, these neuron never "recover" or participate in the representation. To avoid this issue, we used a dynamic threshold that decreases for unresponsive neuron and increases for neurons that are too active. The threshold decreased by $-\epsilon_F$ for a neuron that did not fire any spike in a sliding window of 2.5s, and increased by ϵ_F if its firing rate exceeded 20Hz in the last 2.5s. After about 1000 iterations, the firing rates are always maintained between these two bounds and the thresholds remain constant for the rest of the learning.

In Figures 6,7 the initial recurrent and feedforward weights are drawn from a normal distribution with standard deviations of 0.1 and 0.02 for the feedforward and the recurrent weights respectively; These initial weights are not normalized. The diagonal elements of the recurrent connectivity matrix (the resets) are equal to -0.8. Such strong inhibitory autapses insure the stability of the network in the initial state. In order to speed-up learning we used initially large learning rates ($\epsilon_\Omega = 10^{-2}$, $\epsilon_F = 10^{-3}$) that were progressively decreased to $\epsilon_\Omega = 10^{-4}$ and $\epsilon_F = 10^{-5}$. For re-training to the new non-speech stimulus, we used the learning rates $\epsilon_\Omega = 10^{-2}$, $\epsilon_F = 10^{-3}$. For re-training the feed-forward connections without the lateral connections, we used $\epsilon_F = \frac{1}{4}10^{-2}$).

References

[1] Martin Boerlin, Christian K. Machens, Sophie Denève. *Predictive Coding of Dynamical Variables in Balanced Spiking Networks.* PLoS Comput Biol 9(11) (2013): e1003258.

[2] Ralph Bourdoukan, David G.T. Barrett, Christian K. Machens, and Sophie Denève. *Learning Optimal Spike-based Representations.* Advances in Neural Information Processing Systems 25 (2012).

[3] Peter Dayan and Larry F. Abbott. *Theoretical Neuroscience: Computational and Mathematical Modeling of Neural Systems.* MIT Press (2005).

[4] Christopher J. Rozell, Don H. Johnson, Richard G. Baraniuk, and Bruno A. Olshausen. *Sparse coding via thresholding and local competition in neural circuits.* Neural Computation 20.10 (2008): 2526–2563

[5] Joel Zylberberg, Jason T. Murphy, and Michael R. DeWeese. *A sparse coding model with synaptically local plasticity and spiking neurons can account for the diverse shapes of V1 simple cell receptive fields.* PLoS Comput Biol 7.10 (2011): e1002250.

[6] Pietro Vertechi, Wieland Brendel, and Christian K. Machens. *Unsupervised learning of an efficient short-term memory network.* Advances in Neural Information Processing Systems 27 (2014).

[7] John J. Hopfield. *Neural networks and physical systems with emergent collective computational abilities.* Proceedings of the National Academy of Sciences, 79.8 (1982): 2554–2558.

[8] John Hertz, Richard G. Palmer, and Anders S. Krogh. *Introduction to the Theory of Neural Computation.* Perseus Publishing, 1991

[9] Barak A. Pearlmutter. *Learning state space trajectories in recurrent neural networks.* Neural Computation, 1.2 (1989): 263–269.

[10] David Susillo and Larry F. Abbott. *Generating coherent patterns of activity from chaotic neural networks.* Neuron, 63.4 (2009): 544–557.

[11] Tim P. Vogels, Henning Sprekeler, Friedmann Zenke, Claudia Clopath, and Wulfram Gerstner. *Inhibitory plasticity balances excitation and inhibition in sensory pathways and memory networks.* Science, 334.6062 (2011):1569–1573.

[12] Yin P, Fritz JB, Shamma SA. *Rapid spectrotemporal plasticity in primary auditory cortex during behavior.* J Neurosci. 2014 Mar 19;34(12):4396-408.

[13] Mesgarani N, David SV, Fritz JB, Shamma SA. *Mechanisms of noise robust representation of speech in primary auditory cortex.* Proc Natl Acad Sci U S A. 2014 May 6;111(18):6792-7.

[14] Pengsheng Zheng, Christos Dimitrakakis, and Jochen Triesch. *Network Self-Organization Explains the Statistics and Dynamics of Synaptic Connection Strengths in Cortex.* PLoS Comput Biol 9(01) (2013): e1002848

[15] Andreea Lazar, Gordon Pipa, and Jochen Triesch. *SORN: A Self-Organizing Recurrent Neural Network.* Frontiers in Computational Neuroscience 3 (2009): 23

[16] Wieland Brendel. *On the Structure and self-organized Formation of Neural Population Responses.* PhD Thesis, Ecole Normale Supérieure Paris

[17] Aapo Hyvärinen, Juha Karhunen, and Erkki Oja. *Independent component analysis.* Wiley & Sons (2001).

[18] Bruno A. Olshausen and David J. Field. *Sparse coding with an overcomplete basis set: A strategy employed by V1?* Vision Research 37.23 (1997):3311–3325.

[19] Anthony J. Bell and Terrence J. Sejnowski. *The "independent components" of natural scenes are edge filters.* Vision Research 37.23 (1997): 3327–3338.

[20] Ganguli Deep and Simoncelli P. Eero *Efficient sensory encoding and Bayesian inference with heterogeneous neural populations.* Neural computation (2014).

[21] Michael S. Lewicki and Terrence J. Sejnowski *Learning overcomplete representations.* Neural computation (2000).
