# Supplementary material for: Learning to represent signals spike by spike
Source: PLoS Comput Biol. 2020 Mar 16;16(3):e1007692. doi: 10.1371/journal.pcbi.1007692 (PMC7135338; doi:10.1371/journal.pcbi.1007692)
Supplement: Attachment — Submitted filename: main.pdf [file pcbi.1007692.s003.pdf]

Answer to Reviewers. Learning to represent signals spike by spike

Wieland Brendel Ralph Bourdokan Pietro Vertechi Christian Machens
Sophie Denève

We are grateful to all the reviewers for their comments and constructive suggestions on our manuscript. We are well aware that reading through many pages of equation-heavy supplementary materials is a very time-consuming task, and we thank both reviewers for having gone through the trouble of actually doing that.

We have now revised the manuscript according to the comments and addressed all the concerns raised by the reviewers, as described in detail below. We have also submitted the MATLAB code to reproduce the key figures of the manuscript.

Responses to Reviewer 1

'Learning to represent signals spike by spike' is a normative study on learning rules built to represent multiple signals simultaneously in a spiking neural network. This work starts where previous efforts from the same authors (particularly Boerlin et al. (2013)) had left off. In that previous study, it was shown that a carefully crafted arrangement of synaptic weights allows a network of spiking neurons to represent an arbitrary number of continuous time-dependent signals. These results relied on a precise arrangement of synaptic weights, and the authors had to assume that such an arrangement was given a priori. In the present study, they ask if there exists spike-timing dependent synaptic learning rules to let the network to self-organize to this rather convenient state. Following a normative approached based on a greedy optimization of decoding error, they show that there is a learning rule which can maximize encoding precision and is shows at the same time a voltage and spike-timing dependence in a way that matches, qualitatively, some standard in vitro experiments. The authors reports considerable achievements of their learning rule in spiking neural network (I commend the efforts to establish a Dalean network that self-organizes in precise input representation).

This work comes in an opportune moment as part of the field of computational neuroscience shows a growing interest in learning rules that will ensure that a particular function is conserved. Learning rules have been shown to have a plethora of shapes and properties, and the recent introduction of inhibitory learning rules is only making things worse. There was much focus on rate based learning rules (FORCE learning and its variants), which has recently been shown to work with spiking neurons. In the same vein, there is much recent research on learning weight matrices that are transpose of a known weight matrix in biological implementation of deep learning. All these problems are connected to the present work. Yet the present study is original and distinct from other studies in the sense that it applies to the predictive coding framework and promises of an energy efficient encoding of information.

That being said, I think the paper needs to be revisited carefully in order to unify the narrative, the results presented and and the supplementary material. I expand on my point of view below, but overall I recommend further consideration of this MS for PLoS CB.

We are grateful for the reviewer's positive comments.

1. Abstract. The abstract seems to confuse premises with results. Statements like 'here we show that many single-neuron quantities including voltages... acquire a precise functional meaning' summarizes

the premise of the work rather than the result. Premise in the sense that these are the assumptions from which the main results are derived, but in that case also because these are the results of a previous paper from the same group. Similarly for the conclusion sentence of the abstract. Going a little further, the question of finding THE level at which THE functional meaning emerges is not a key question in neuroscience. There are multiple levels of description and therefore functionality has multiple levels of description. Multiple levels of description, but also multiple types of systems with membrane potentials (the spike-based predictive coding framework does not apply to non-spiking retina despite the shared coding and energy constraints). These statements is made even more out of place when we consider the fact that I don't think the work presented in the MS addresses this question. The work is about whether biological-looking learning rules can give rise to the nice benefits of the predictive coding framework. In effect, it would be nice if the abstract would be more to the point. The introduction is good, so just a condensed version of the intro would do. Similar issue with the end of the discussion.

We agree that the abstract may have been too broadly worded. We now rewrote parts of the abstract to better identify the focus and novelty of this particular paper.

We still like to point out, though, that voltages (and other biophysical quantities) acquire a specific functional meaning after learning. The reviewer is correct to point out that this is very much by design. However, the fact that voltages do represent coding errors after learning is fundamental to the type of network architectures we study, and yields an important functional bridge between single-neuron biophysics and the population code. (Classical) models of spiking neural networks do not treat voltages in this way. (That said, there are, of course, single-neuron models, such as the ones by Urbanczik and Senn, that do, which we now point out in the discussion.)

2. Intro. I thought I would mention a few related works that I think are germane to the present study:

- Membrane potential as prediction error: Urbanczik and Senn, Neuron (2014).
- Learning the transpose of weights: Burbank (2015) uses an STDP setup to do so.
- Further learning of the transpose of the weights in rate models; Akrout et al. (2019); Lansdell, Prakash and Kording (2019)

Thanks for pointing out these references. We have included them in both the introduction and discussion. In the introduction, we now clarify better that our work focuses on unsupervised learning, and we now mention that similar problems exist in supervised learning:

> In turn, the derivation of learning rules under locality constraints has often relied on heuristics or approximations (Zylberberg et al, 2011; Savin et al, 2010, Bourdoukan et al 2012, Burbank et al, 2015), although more recent work has shown progress in this area (Vertechi et al, 2014; Pehlevan et al, 2015; Pehlevan et al, 2018). We note that supervised learning in neural networks faces similar problems, and recent work has sought to address these issues (Whittington et al, 2017, Guerguiev et al, 2017, Sacramento et al, 2018, Akrout et al, 2019, Lansdell et al, 2019). We will here focus on unsupervised learning.

In the discussion, we now write:

> In the future, these ideas may be combined with explicit single-neuron models (Urbanczik et al, 2014) to turn local learning rules into global functions.

3. Decoder weights. In many places the decoder weights are said to be unknown, but then they are the target of the recurrent weights, later they are the target of feedforward weights. How can the weights be targets without being known? Similarly, the decoder network is sometimes an explicit network elements, but recurrently it is just a virtual presence introduced for the sake of argument. It was particularly confusing in the supplementary materials: D is assumed unknown but should follow S.23,

which is in effect F. Then F is assumed unknown, but derived to be D. I am left with the impression that there is a circular argument in the learning of F with D that is not fixed a priori. In my point of view, the circular argument is present in sections like 8.2 of supplementals. Same with section 6, which (6.4 has F to mimic D, but 6.5 chooses D with F).

We thank the reviewer for helping us to clarify this concept. The reviewer is correct that $\mathbf{D}$ is unknown before learning. Instead, over learning the recurrent weights $\mathbf{\Omega}$ converge to a low-rank configuration of the type $\mathbf{\Omega} = -\mathbf{FX}$ where $\mathbf{F}$ is the known feedforward matrix and $\mathbf{X}$ is some a priori unknown matrix. It turns out, however, that the matrix $\mathbf{X}$ is actually the optimal decoder to reconstruct the signal (see Section 6.5), and so we always identify the matrix $\mathbf{X}$ as the decoder $\mathbf{D}$ throughout the manuscript, even though the decoder is unknown before learning and is only a quantity that can be computed from $\mathbf{\Omega}$ and $\mathbf{F}$ after learning. (See also Section 6.2 in the supplementary Material).

In other words, the decoder matrix $\mathbf{D}$ is unknown before learning and can only be inferred from $\mathbf{\Omega}$ after learning by solving the equation $\mathbf{\Omega} = -\mathbf{FD}$ for $\mathbf{D}$. We emphasize that this decoder $\mathbf{D}$ is still not explicitly present in the network, only implicitly. Still, it is mathematically defined, and so we can now state that the learning of the feedforward weights $\mathbf{F}$ needs to converge to $\mathbf{D}$.

We have revised parts of Chapter 6 and Section 8.2 to clarify these points.

4. Supplementary material. I could not fully follow the supplementary material. There was too much back and forth between different formalisms and different sets of assumptions. Current based learning, then voltage-based learning, then L1-L2 costs, then summary of some of it, there has to be a more streamlined version. There are a few, perhaps interesting, theoretical results that are not part of the results as far as I can see. Particularly parts of section 5.

We agree with the referee that the supplementary material are quite long and very technical. When writing the manuscript, we decided to write a main paper that contains the key ideas with a minimum of math, so that all readers, including those without a strong quantitative background, can get the gist of the work. At the same time, we felt that we should provide all of the technical details in the Supplementary materials, so that really interested readers can follow through all the details.

Given this split, we have then decided to refer to technical details from the results section point by point, rather than making the supplementary materials a second, more detailed and technical version of the main paper. In turn, these choices have led to a supplementary material that is divided into many different sections, rather than being a coherent text on its own.

The reviewer is correct that this organization of the material has certain drawbacks, and a natural solution may seem to move some of the materials into the main text and in turn shorten and condense the supplementary materials. The way we see it, however, is that each re-organization has its own advantages and disadvantages. We therefore prefer to keep the overall organization of the manuscript.

5. Figure 2 does not give enough credit to learning recurrent connections. As we can see in Fig. 2A, the error goes down dramatically at then end of the recurrence-learning, so the signal reproduction is near perfect before learning the FF weights. Since it is not clear in Figure 3 whether the recurrent weights have been adequately learned, this brings me to the question eluded to earlier: can you prove that FF learning is essential? In which case?

In Fig. 2, after recurrent learning, even though the mean firing rate (in red) is much reduced, there is still some error (in blue) left. In this low-dimensional example, learning of the feedforward weights is required to ensure that the feedforward weights span the whole input space. Indeed, a network with feedforward weights as in Fig. 2B, left panel, will struggle to encode inputs in the bottom right and top right quadrants, *even after* the recurrent synapses have been learnt.

More generally speaking, even if the average error is already quite reduced after learning the recurrent weights, a network may still exhibit large errors for *some* input signals. In other words, learning to be excellent on average is not the right metric—rather, the network should be excellent for

all input signals. Learning of the feedforward weights is therefore required to make sure that all signals are represented with high quality.

6. Figure 4 shows inverted I-E connections with respect to EE, but the text mentions the learning rules are the same. Also, on which side is $t_{post} > t_{pre}$ in the learning windows?

The x-axis in the learning windows is $\Delta t = t_{\rm pre} - t_{\rm post}$. There does not seem to be any strict convention in the field, and we have added an explanation to the legend in order to be crystal-clear.

The learning rules for the EE synapses are indeed the same, but there is an additional sign-flip we need to take into account when mapping the learning rules to the experimental STDP protocols. For a single synapse, the learning rule $\Delta\Omega = -2V + \Omega$ essentially states that Ω will become more negative when the postsynaptic voltage was depolarized, and it will become more positive when the postsynaptic voltage was hyperpolarized.

For an inhibitory synapse, becoming more negative means that the synapse is strengthened. For an excitatory synapse, however, becoming more negative means that the synapse is weakened. Consequently, when we map our learning rules on STDP-type protocols, we need to flip the sign on the weight changes for the excitatory synapses.

We have added some explanatory text to the results section to clarify these points:

> The insets show cartoon illustrations of the learning rules, stemming from STDP-like protocols between pairs of neurons, with the x-axis representing the relative timing between pre- and postsynaptic spikes ($\Delta t = t_{\rm pre} - t_{\rm post}$), and the y-axis the change in (absolute) weight. Note that increases (decreases) in synaptic weights in the learning rules map onto increases (decreases) for excitatory weights and decreases (increases) for inhibitory weights. This sign flip explains why the STDP-like protocol for EE connections yields a mirrored curve.

7. x is assumed to white at many places, but it is simulated as a non white signal (section 14.1 and eq. S.1)

For the sake of simplicity, the learning rules are initially presented and simulated in the setting of white signals (Figures 2, 3A,B, and 4, and also Figure 8). However, it is possible to generalize the network architecture and plasticity to handle non-white signal by introducing the input covariance matrix in the loss function (Supplementary Sect. 12). This modifies the learning rule for the feedforward weights (see Figure 3C,D). These modified learning rules are used in Figures 5,6, where we learn a network architecture for speech signals (which are non-white). We now explicitly point out, when introducing Figure 2, that the input signals are uncorrelated.

8. Having filtered x and non-filtered x denoted by the same variables in the main results section is disturbing, please fix.

We thank the reviewer for pointing out the inconsistency in notation between main text and supplementary material. We now introduce a separate notation for currents also in the main text (on page 4):

> Each neuron's membrane potential is driven by feedforward input signals, $x_j(t)$, which we will model as a leaky integral of input currents $c_j(t)$, and by recurrent inputs, that feed the output spike trains, $o_k(t)$, back into the network.

We have updated eq. 1 accordingly.

9. Please explain why noise is included in the simulations. I presume it is required to some extent.

The role of noise is two-fold. First, it showcases an important feature of our networks' architecture: even though the signal decoding is robust, small voltage fluctuations can cause large differences in the spike trains of single cells (see Figure 2E,F after learning; see also Boerlin et al, 2013, PLOS CB).

Second, this spike train variability allows us to approximate the network activity in the limit of large network, which is necessary to give convergence guarantees for the voltage-based learning rule, see pages 26-27 of the Supplementary Material:

> For large networks, however, even small noise sources will considerably randomize the timings and sequences of spikes (Boerlin et al., 2013), and so in this limit it makes sense to analyse the fixed points under the assumption that spikes are distributed according to an inhomogeneous Poisson process with mean firing rates $\bar{r}_k(\mathbf{x})$.

10. Please verify the transpose on Eq. S.12 and S.10.

Following our conventions, we define the decoder $\mathbf{D}$ as the $M \times N$ matrix mapping the N-dimensional activity $\mathbf{r}$ onto the M-dimensional estimated signal $\hat{\mathbf{x}}$. In turn, we define the $N \times M$ feedforward matrix $\mathbf{F}$ as mapping M input dimensions onto N neurons.

Given these definitions, we indeed require that the feedforward matrix approximates the transpose of the decoder matrix at the end of learning, and so $\mathbf{F} = \mathbf{D}^\top$.

11. L* not defined in first equation of Section 4 of supplementary materials. Wording is a bit confusing just before that equation as it is as if a loss function is defined by the equation. I find this equation and the one in 5.1 confusing. The goal is not to determine L*, but to determine D or o that achieves L*. Why not use argmin?

We thank the reviewer for pointing out the confusing phrasing. We now use a more straightforward formula, see Supplementary Sect. 4:

> Specifically, we will seek to find the set of spike trains, $\mathbf{o}(t)$, that minimizes the loss function
>
> $$L(\mathbf{o}) = \left\langle \|\mathbf{x} - \mathbf{D}\mathbf{r}\|^2 + \mu \|\mathbf{r}\|^2 + \nu \|\mathbf{r}\|_1 \right\rangle,$$
>
> where the average in angular brackets is taken over all possible spike trains.

and Supplementary Sect. 5.1:

> In short, we want to determine the best possible decoder $\mathbf{D}$ for a given network, i.e., minimize
>
> $$L(D) = \left\langle \|\mathbf{x} - \mathbf{D}\mathbf{r}\|^2 \right\rangle, \quad (1)$$
>
> where $\mathbf{r}$ is the filtered spike train output of the network.

12. What is a population spike in (section S7)

We refer to a spike emitted by any neuron in the population. We now introduce this notion at the beginning of Supplementary Sect. 7 on page 15, together with the notion of *population interspike interval*:

> The shortest possible time-scale at which a single spiking neuron can be balanced is limited by the interval between any two consecutive *population spikes*, i.e., spikes emitted by any neuron in the population.

13. Main results section p14-15. The term decoding weights has been used instead of FF weights.

We have corrected the mistake and also made sure to explain the concept of the decoding weights better (see also response to comment 3).

14. Note that FF weights from thalamus are fixed after critical period.

There is certainly a lot more plasticity during the critical period. However, we do not really think that there is any evidence that the thalamo-cortical synapses are truly 'fixed' once the critical period is over. Hence, we believe that our predictions make sense even after the critical period is over.

15. I was a bit frazzled by the overly simple descriptions

Our general aim was to make the manuscript as readable as possible for a more biological audience, and to move the technical and more complex aspects of the work to the supplementary materials. That is certainly not ideal for the more theoretically-minded reader, but we see it as a reasonable compromise, given the mixed audience the paper will likely face.

Responses to Reviewer 2

The manuscript addresses efficient coding of inputs by spiking neural networks. Theories of efficient coding have a long history in neuroscience. The approach taken here differs from most other works in the assumptions made on the generation of spikes. In the proposed scheme, spikes of individual neurons may appear noisy and stochastic, but in fact every spike is generated by a rule that aims to minimize the reconstruction error of an implicit linear decoder, while taking into account the activity of all others neurons in the network.

The above approach is related to previous works from the same senior authors, in which the goal was to design recurrent networks of spiking neurons that precisely implement certain dynamics. The previous works argued that the emergent solution is one in which internal variables related to the dynamics will be efficiently represent by spikes. In this sense, the present work takes a step backwards, by focusing solely on efficient coding of an external variable. In my opinion this apparent step backwards is in fact very useful, as it allows for a more rigorous treatment of this family of problems.

I appreciated the detailed supplementary text which, despite being highly mathematical in nature, lays out the ideas in a principled and usually clear manner. The exposition of the ideas in the main text, in comparison, is sometimes too vague or sketchy to be comprehensible. There are some aspects of the presentation that can be improved in the supplementary text as well.

My overall assessment is that this work offers an important contribution to the theory of neural coding, and its content is therefore very appropriate for publication in PLoS Computational Biology. However, there are some issues that require additional explanation or correction.

We thank the reviewer for the appreciation of our work.

General comments

1. It will be helpful to explain more explicitly how this work differs from previous works by the same authors on optimization of spiking neural networks (Refs 21-24). My understanding is that the main differences are: (a) in the goal (as mentioned above), (b) in the training scheme, i.e. in proposing an online learning rule which is biologically plausible. Even though (b) is stated in the introduction, it is not sufficiently clear from the phrasing that this is the key contribution of the present manuscript.

The referee correctly points out that the novelty of the work lies in the training scheme. In previous publications (Refs 21-24), the structure of the network was simply assumed. (More precisely, a fixed decoder was assumed, and the structure of the optimal network was derived from this assumption.) In turn, our previous work had no training scheme, and it was a priori unclear whether it was possible for the network to self-organize into the desired structure via biologically-plausible local learning rules. The current manuscript now shows how this can be done. We have reworded the abstract to clarify better the novel aspect of our study (see also response to comment 1 from Referee #1).

In terms of the optimization goal (regardless of the training scheme), it will be helpful to understand whether the goal considered here is a special instance of the problem considered in the previous works. If so, what is the relationship between the solutions for synaptic connectivity obtained in these different works (even if the learning rules are different).

In our previous work, the decoder was simply given by a putative downstream readout, and the optimal recurrent and forward connectivity were determined through this downstream readout. In the current manuscript, we first define an optimal decoder (defined as the putative downstream readout that would minimizes the time-averaged reconstruction error across all possible network architectures), and then show how to learn the network architecture for this optimal decoder.

A specific, somewhat technical difference between the previous works and the present manuscript is that in the former works, the dynamics involved both slow synapses and fast (or instantaneous) synapses, whereas here all synapses are instantaneous - see also comment 4. It will be helpful to comment on this.

The referee is correct that the present manuscript has no slow connections. Indeed, we consider the learning problems for the slow and fast connections separate learning problems. Loosely we think of the slow connections as a supervised learning problem (see e.g. Alemi, Machens, Deneve, Slotine, 2018, AAAI Conference), and the fast connections an unsupervised learning problem (this paper).

2. Likewise, the manuscript proposes two learning algorithms and argues that both are optimal. Do they converge to the same solutions, or at least the same values of the objective functions? If the solutions are not the same, is this because there are multiple local minima? or because of some other reason, such as lack of optimality?

One of the reasons for a need to address these questions is that the argumentation for convergence to optimal solutions with the voltage-based rules relies on several uncontrolled approximations and assumptions. Hence, numerical testing of its performance relative to other schemes is warranted.

Both learning rules are based on the same objective function, i.e.

$$L(\mathbf{o}) = \left\langle \|\mathbf{x} - \mathbf{D}\mathbf{r}\|^2 + \mu \|\mathbf{r}\|^2 + \nu \|\mathbf{r}\|_1 \right\rangle,$$

as mentioned in the Supplemental Material, page 9. However, they do so via different *proxy* loss functions. As shown in Supplementary Sects. 7 and 9, both learning rules have the architecture $\mathbf{\Omega} = -\mathbf{F}\mathbf{D} - \mu\mathbf{I}$, and $\mathbf{F} = \mathbf{D}^\top$ as fixed points.

The referee correctly points out that this leaves two open problems. First, are the fixed points local or global optima of the loss function? Second, what is the nature of the matrix $\mathbf{D}$ after convergence?

Concerning the first question, Supplementary Sections 9 and 10 have convergence proofs showing that the fixed points are global attractors of the learning rules. The referee is correct, though, that these proofs are not airtight for all possible scenarios. In practice, however, i.e., in simulations of networks with (low-dimensional) input signals, we have always found convergence to the global optimum. Figure 2B shows an example. (Networks with higher-dimensional input signals have been harder to learn for numerical reasons—the simulation times become prohibitively long on desktop computers.)

Concerning the second question, we show in Supplementary Sects. 8 and 10 that both learning rules achieve $\mathbf{F} = \mathbf{D}^\top$ and optimize $\mathbf{D}$ with respect to the efficient coding objective. That said, we cannot ensure that the final values of $\mathbf{D}$ coincide for both learning rules in numerical simulations, as the optimum of the efficient coding objective function can be quite shallow. Moreover, unlike in classical efficient coding, here the decoder $\mathbf{D}$ is not renormalized (even though the learning rules ensure that $\|\mathbf{D}\|$ does not diverge to $+\infty$). The techniques used to prevent $\|\mathbf{D}\|$ from diverging can in general lead to different absolute values of $\mathbf{D}$ depending on the learning rule chosen.

Nonethess, we have always found overall similar values of the loss function after learning in simulations.

3. The relationship to other works on efficient coding is not sufficiently clear, and is sometimes confusing. Most works on efficient coding focused on the receptive fields of single cells, and not on how the recurrent connectivity can generate jointly an efficient representation by spikes. Accordingly, spiking was mostly considered as generating noise which is independent in different neurons. In this respect, the present work offers an important advancement.

On the other hand, the present work does not incorporate important lessons that were learned from works that focused on the optimal receptive fields in a population of neurons that encode a multidimensional input:

a First, some works on efficient coding considered mutual information rather than reconstruction error as the optimization goal.

b Under certain variants of optimization with sparseness constraints, optimal solutions implement ICA on the inputs, not PCA. The solutions obtained here are only sensitive to the covariance matrix of the inputs and therefore do not implement ICA.

c Under L2 constraints and without noise in the inputs, networks typically implement whitening without the need for a modified loss function as in Section 12. Here (in the present work) it seems that the whitening is forced into the solution by the choice of the loss function, and this requires some clarification.

d Once noise is included in the inputs, it is typically suboptimal to simply whiten the inputs. This was clarified for example by the classical works of Attick and Redlich on encoding of visual stimuli by the retina, as well as others.

It is possible that the formulation proposed in the present work can be extended to incorporate all these ingredients, but as far as I can see this is not the situation right now. Therefore, it will be helpful to lay out more clearly and correctly the relationship to previous works on encoding of multidimensional inputs and acknowledge the limitations of the present work. The last paragraph of supplementary section 1.4 is very confusing in this respect.

We thank the reviewer for this interesting remark. The last paragraph of supplementary Section 1.4 gave the material a bit short shrift, and we have now expanded this paragraph to better explain the scope of our work.

Briefly, the term 'efficient coding' has been used in various ways in the past, as correctly pointed out by the reviewer. We use the term efficient coding in two senses: First, we simply want to express that the spike code generated by our networks is far more efficient than classical codes such as Poisson codes. Here, we measure efficiency as number of spikes fired for a given accuracy of the code. Second, we can understand efficient coding in our work in the sense of Olshausen and Field (1996), where efficiency was equated with sparsity. The loss function used by Olshausen and Field is mathematically identical to our loss function, and also relates this work to ICA. More specifically, it relates our work to overcomplete ICA, as explained in e.g. Lewicki & Sejnowski, 2000, Neural Computation, 12:337–365.

Concerning point (a), we now explain better that we are not using a general, mutual-information based approach, but one based on mean-square errors. Specifically, we now write at the beginning of the discussion:

> We have measured efficiency with an objective function that combines the mean-square reconstruction error with various cost terms. While mathematically simpler than mutual-information-based approaches, our objective function includes both principal and overcomplete independent component analysis as special cases.

Concerning point (b), we have now clarified that ICA can be achieved in our networks via a sparsity cost, just as in Olshausen and Field (1996). We added 'overcomplete' when refering to ICA in the main text (see above), and in Section 1.4 of the Supplementary Material, we now write:

> We note that algorithms such as principal component analysis (PCA) and independent component analysis (ICA - Hyvärinen et. al, 2001) can be formulated as special cases of this general class of learning problems. In particular, Lewicki and Sejnowski (2000) show how ICA relates to efficient coding in the presence of a sparsity cost.

Accordingly, while our work does not relate to all possible ICA algorithms, it does relate to overcomplete (or sparse) ICA, because it shares the same loss function.

Regarding point (c), the change in the cost is not so much about whitening as it is about invariance with respect to linear transformations of input signals. As the recurrent learning rules are local, they have no access to transformations in the input (which is only accessible after a linear transformation $\mathbf{F}$). The covariance structure of the input allows us to define a loss that is not affected by such transformations: as an added benefit, the network is now able to efficiently encode non-white signal. We now mention the invariance considerations more explicitly in the Supplementary Material on page 31:

> [...] we modify the loss function,
>
> $$\ell = (\mathbf{x}_c - \mathbf{Dr})^\top \mathbf{C}^{-1} (\mathbf{x}_c - \mathbf{Dr}) + \mu \|\mathbf{r}\|^2 + \nu \|\mathbf{r}\|_1$$
>
> where $\mathbf{x}_c = \mathbf{x} - \bar{\mathbf{x}}$ is the mean signal and $\mathbf{C} = \langle \mathbf{x}_c \mathbf{x}_c^\top \rangle$ is the signal covariance. It is important to note that the more general loss (S.43) is invariant with respect to linear transformations in the input $\mathbf{x}$. If the input signal is replaced by a transformed signal $\mathbf{x}' := \mathbf{Mx}$, with $\mathbf{M}$ an invertible matrix, the loss function is not affected. It is sufficent to replace $\mathbf{D}$ with $\mathbf{MD}$ to recover an equivalent optimization problem.

Regarding point (d), that is an interesting point for future work, but we think it goes beyond the scope of our study, which is primarily about learning rules in a spiking network.

4. The learning of feedforward connections is chosen to be slow compared to the learning of recurrent connections, and this is apparent already in Figure 2 but not discussed (unless I missed this). Later on, in at least two instances, the difference in time scales is mentioned as an important requirement or consequence, and time scale separation is used in some of the argumentation in the supplementary material. However, as far as I understand, the results don't demonstrate that successful learning occurs only if the time scale of feedforward learning is slow compared to the recurrent learning, and it will be helpful to comment directly on this question.

We thank the reviewer for pointing out this omission. When first discussing the learning of the feedforward weights in the context of Figure 2, we now write:

> The effect of the feedforward plasticity rule is shown in Fig. 2Aiii–Giii. The feedforward weights change slowly until the input space is spanned more uniformly (Fig. 2Biii). While these changes are occurring, the recurrent weights remain plastic on a faster time scale and thereby keep the system in a balanced state.

The rationale is that, once the recurrent learning brought the network to the state $\mathbf{\Omega} \approx -\mathbf{FD}$, it is important that the feedforward learning does not disrupt this factorization. We now explain this in Supplementary Sect. 6.4.

> The network architecture that we have derived so far still deviates from the optimal architecture, since $\mathbf{F}$ and $\mathbf{D}^\top$ are not necessarily the same matrices. Accordingly, we need to somehow make sure that the feedforward weights $\mathbf{F}$ align with the (unknown) decoder $\mathbf{D}^\top$. This update of the feedforward connections should happen at a slower time scale than recurrent learning, to ensure that the equation $\mathbf{\Omega} = -\mathbf{FD} - \mu\mathbf{I}$ is approximately preserved.

We also note that if the time scale of the feedforward learning is too rapid, the learning rules no longer converge, as suggested by our mathematical derivations, and as observed in many numerical simulations.

More specific comments

5. In the network dynamics (Eq. 1) synaptic currents are instantaneous. This should be stated explicitly. The phrasing in page 16 misrepresents the situation, since the postsynaptic current generated by an incoming spike is a delta function, not a jump followed by exponential decay. The *voltage* jumps and decays exponentially following a spike, not the *current*. This dynamics of the voltage has nothing to do with synaptic dynamics.

We thank the reviewer for pointing out the inaccuracy. Indeed the term *current* is incorrect here: we intended to refer to *voltage* and postsynaptic *voltage* dynamics. We have now corrected the text on page 17:

> A final concern could be to what extent the learning rules rely on overtly simplistic synaptic dynamics—each spike causes a jump in the postsynaptic voltage followed by an exponential decay.

We now also mention explicitly, on page 4, that the recurrent synapses are instantaneous in (Eq. 1):

> For simplicity, here we consider *instantaneous* synaptic transmission: the impact of synaptic delays on the network will be examined in Fig. 7.

6. I recommend to briefly mention the basic requirements from an optimal decoder in the main text, before discussing the current and voltage based learning rules. These requirements are currently derived in sections 2-5 of the supplementary material and summarized in section 6. It will be very helpful to clearly lay out the requirements in the main text, preferably using some equations. Otherwise, it remains unclear while reading the main text alone that optimization of the error entails certain concrete requirements on the recurrent and feedforward connectivity and (implicitly) on the decoder. Consequently, it is not clear to the reader in what sense the local learning rules derived later achieve optimal performance.

We can see the merit in what the reviewer is suggesting, i.e., to essentially provide more mathematical details in the main text. However, we prefer to have a stronger split between intuitions with 'light math' in the results and then the more technical parts in the Appendix. We completely agree that this organization puts a bit of burden onto the more theoretically inclined audience, but we think that it's worth the benefit of being more comprehensible for more experimentally oriented neuroscientists.

That said, the reviewer is correct that we never explicitly mentioned that the decoder will also be optimized. In the main text, we have corrected that omission and now write (beginning of results),

> The second objective of the network will be to find, among all possible spiking outputs, and all possible decoders, the ones that are the most efficient.

7. The discussion on the current based decoder lacks concreteness in the main text. It will be helpful to provide some equations involving g, in similarity to Eqs. (5) and (6) that are provided for the voltage based rule. Otherwise it is not clear that the idea of balancing the currents translates into a specific synaptic learning rule. It will also be helpful to refer to the appropriate supplementary sections earlier in the text.

We intentionally kept the mathematics of the current-based learning to the supplementary materials. Our aim to discuss the current-based learning rules in the main text was two-fold. First, the idea of current-based learning provides nice intuitions. Second, having two learning rules shows that the learning rules leading to the optimum are not unique. We believe that is an important insight.

We prefered to keep the math of the current-based learning rules in the supplementary materials so as to not stretch the main text too long or make it too technical. We agree with the referee, though, that a better reference to the supplemental sections will be helpful. We now write (before the introduction of the learning rules):

> We developed two ways of reaching the balanced regime (see Supplementary Materials S6 for a high-level, technical overview). The first scheme balances excitatory and inhibitory currents on a fine time scale (see Supplementary Materials S7 and S8 for details), while the second scheme minimizes the voltage flucutations (see Supplementary Materials S9–S12 for details). We here briefly explain the current-based scheme, but then focus on the voltage-based scheme for the rest of the text.

8. Likewise, it will be helpful to mention what are the relevant supplementary sections early on when introducing the voltage based learning, to clarify that there is a systematic derivation behind the proposed rules, which are only qualitatively motivated in the main text.

We agree - please see above.

9. The argumentation at the top of page 9 is difficult to follow and seems circular. The problem starts with "Since the connectivity structure dictates that the voltage becomes a function of the global coding error": isn't this part of what needs to be shown? How does this follow from the stationary state arising from rule (5)?

Here we are only showing that $\mathbf{\Omega} = -\mathbf{FD}$ is a fixed point (for *some* matrix $\mathbf{D}$), we are not showing that the learning rules converge to that fixed point. (A convergence proof is provided in the supplementary materials.)

To show that $\mathbf{\Omega} = -\mathbf{FD}$ is a fixed point, one only needs to show that the net changes in $\mathbf{\Omega}$ vanish, i.e., that $\langle d\Omega_{ik}/dt \rangle = 0$. To show this, we first use the fact that, whenever $\mathbf{\Omega} = -\mathbf{FD}$, the voltage becomes a function of the coding error, as shown in Eq. 4. Plugging this equation into the learning rule, then shows that the average derivative vanishes. We now make the logic more clear in the text on page 9:

> Since, whenever $\mathbf{\Omega} = -\mathbf{FD}$, the connectivity structure dictates that the voltage becomes a function of the global coding error, as stated in Eq. 4, the stationary point can be rewritten as $\Omega_{ik} = -2\sum_j F_{ij}\langle x_j - \hat{x}_j\rangle_k$.

The last equation then shows that the stationary point is of the form $\mathbf{\Omega} = -\mathbf{FD}$, and the derivatives will vanish if we identify the decoder $D_{jk} = 2\langle x_j - \hat{x}_j\rangle_k$.

10. If I am not mistaken, the exposition in the main manuscript fails to acknowledge the need to show that the rules converge to a decoder that minimizes the loss function (Sections 8.3 and 10.2). This is something that I was puzzled about while reading the main text, and became clear only while reading carefully through the supplementary text. I recommend to mention this explicitly.

We thank the reviewer for pointing out this omission. We now write (on page 13):

> More specifically, the feedforward weights have become identical to the decoding weights, $F_{ik} = D_{ki}$, and the latter minimize the objective function, Eq. 3.

11. Fig. 4 caption: the text on panel A discusses an STDP rule depending on the timing of pre- and post- synaptic spikes. However the recurrent learning rule discussed in the text involves the presynaptic spike and the postsynaptic voltage, while the feedforward rule involves a postsynaptic spike and the presynaptic continuous input. Please clarify.

In a previous section of the manuscript, on page 8, we refer to Clopath et al. (2010), which propose multiplication of presynaptic spike and postsynaptic voltage as a *mechanism* for STDP.

> [...] According to this rule, the recurrent connections are updated only at the time of a presynaptic spike, and its weights are increased and decreased depending on the resulting postsynaptic voltage. While this rule was derived from first principles, we note that its multiplication of presynaptic spikes and postsynaptic voltages is exactly what was proposed as a canonical plasticity rule for STDP from a biophysical perspective (Clopath et al., 2010).

The objective of our analysis is to argue that, even though our rules are defined in terms of postsynaptic voltage, simulations show that they exhibit STDP-like feature. We have now clarified this in the discussion, on pages 21/22:

> As a result, even though our proposed learning rules are not defined in terms of relative timing of pre- and postsynaptic spikes, most connections display some features of the classic STDP rules, e.g., LTP for pre-post pairing, and LTD for post-pre pairing (Caporale and Dan, 2008; Feldman, 2012).

12. The results on more realistic synaptic dynamics (page 16) are not sufficiently clear and detailed.

   a Please explain clearly that the synapses were instantaneous up to that point (see also comment 1).

   b The phrasing "transmission delays" is confusing, as it suggests a simple delay of the synaptic current without any other modification in its structure. A better phrasing will be something along the lines of "realistic, non-instantaneous synaptic currents" with reference to panel D of Figure 7.

   c In panel C, the ratio between the error with 2ms delay and 0 ms delay is very large, more than an order of magnitude. In what sense then is the degradation in performance small, as stated in page 16? And how does the error compare to its value at the beginning of training? Does it still improve with increase of the network size?.

Concerning item (a), we now explicitly mention that the synapses are considered instantaneous - see response to point (5).

Concerning item (b), we agree that the wording was confusing. We are now more explicit in our definitions and write:

> To address this question, we also simulated the network assuming more realistic synaptic dynamics (Fig. 7D). We measures the effective delay of transmission as the time-to-peak for a postsynaptic potential.

Concerning item (c), we eliminated the word 'small'. We emphasize that the performance of the networks improves even in the delayed case. However, the overall improvement is simply smaller.

13. The Pseudo-Code in the supplementary text may be helpful for reproducing the results. It is less useful if one wishes to simply understand in mathematical terms what were the dynamics of neurons and synapses in each simulation. It's difficult to do so now because equations are scattered in the main manuscript and in the multiple sections of the supplementary material. Therefore, I suggest to add a summary of the equations used, either in the supplementary text or as a methods section. Either repeat the equations or refer to them by number, but specify clearly what were the equations that were used in each Figure.

We agree with the reviewer that the results and figures should be easily reproducible, and that the current explanations and pseudo-code are unlikely to achieve that job. We believe that the best way to do so is to simply share the MATAB code for all relevant figures, which we have now done, and will do upon publication of the paper.

14. I didn't fully understand the logic of Sec. 5.2. I understand that greedy optimization of the spike timing yields Eqs. S.10 and S.11. But if the threshold is instead fixed at some value that deviates from optimality, is it obvious that the best choice for the decoder weights is the one that obeys these two equations?

In Eq. S.11, we assume that the decoder length $\|\mathbf{D}_n\|$, and the linear and quadratic costs, ν and μ, are known, and we calculate the threshold. In Eq. S.15, we assume that the threshold is known, as are the linear and quadratic costs, and we then calculate the decoder length.

These choices are not so much driven by optimality considerations, but rather by the consideration to keep things simple. In principle, one could freely choose the threshold and decoder length. This would be equivalent to giving a separate linear cost, ν_n, to each neuron's firing rate, i.e., to weighting the cost of each spike differently. We refrain here from doing so, as it would make things more general, but without adding conceptually new insights.

15. In page 19 (section 8.3), at the left hand side of the equations, why is there no average symbol on the voltages? In addition, the argumentation is confusing since $\langle V \rangle$ is assumed to be close to zero, but then naively $\langle \Delta V \rangle$ will remain close to zero as well. I suppose that all terms in the equation are small but in the last line of the equation $\langle V \rangle$ can somehow be shown to be small compared to the first term. This is not done in a sufficiently convincing manner.

We thank the reviewer for signaling the inaccuracy. We refer to the average voltage difference $\langle \Delta \mathbf{V} \rangle$. The reviewer is correct in pointing out the confusion concerning $\Delta \mathbf{V}$. We were originally referring to the change in voltage due to feedforward learning assuming that the firing rates do not change. We now instead consider explicitly a change in firing rates, in which case both $\mathbf{V}$ and $\Delta \mathbf{V}$ will be small. This is now explained more clearly in the Supplemental Material on page 19:

> To keep things simple, we will assume that the recurrent connectivity is already learnt, and we will write $\Delta \mathbf{F} = \lambda(\mathbf{D}^\top - \mathbf{F})$ for the feedforward update, where λ is a small feedforward learning rate. Let $\Delta \mathbf{r}$ be the corresponding change in firing rate. The resulting change in the voltage will then—on average—be proportional to

$$
\begin{aligned}
\langle \Delta \mathbf{V} \rangle &= \langle \Delta \mathbf{F}(\mathbf{x} - \mathbf{D}\mathbf{r}) + \mathbf{\Omega} \Delta \mathbf{r} \rangle \\
&= \lambda \langle (\mathbf{D}^\top - \mathbf{F})(\mathbf{x} - \mathbf{D}\mathbf{r}) \rangle + \langle \mathbf{\Omega} \Delta \mathbf{r} \rangle \\
&= \lambda \langle \mathbf{D}^\top (\mathbf{x} - \mathbf{D}\mathbf{r}) - \mu \mathbf{r} \rangle - \lambda \langle \mathbf{F}(\mathbf{x} - \mathbf{D}\mathbf{r}) - \mu \mathbf{r} \rangle + \langle \mathbf{\Omega} \Delta \mathbf{r} \rangle \\
&= \lambda \langle \mathbf{D}^\top (\mathbf{x} - \mathbf{D}\mathbf{r}) - \mu \mathbf{r} \rangle - \lambda \langle \mathbf{V} \rangle + \langle \mathbf{\Omega} \Delta \mathbf{r} \rangle.
\end{aligned}
$$

Since the learning of $\mathbf{F}$ happens on a much slower time-scale than the learning of the recurrent weights, both the l.h.s, i.e. the average voltage difference, and the second term on the r.h.s., i.e., the average voltage, will be close to zero, as the network remains in a tightly balanced state throughout the learning of the feedforward weights. As a consequence the change in firing rates approximately respects the following equation

$$\begin{aligned} \langle \mathbf{\Omega} \Delta \mathbf{r} \rangle &\approx -\lambda \langle \mathbf{D}^\top (\mathbf{x} - \mathbf{D}\mathbf{r}) - \mu \mathbf{r} \rangle, \\ &= \lambda \frac{\partial L}{\partial \mathbf{r}} \end{aligned}$$

where L is the averaged loss function in the absence of the linear cost term.

16. In the last paragraph of page (19) of the supplementary material I did not understand the argument. If r (not $\langle V \rangle$) would change in the direction of the anti gradient with respect to r, the argument would be clear.

We thank the reviewer for pointing out this unclear passage in the proof. As mentioned in the previous reply, we now consider the induced change in firing rate explicitly and show that, assuming stability of the network and therefore that the recurrent inhibitory connections are a negative definite matrix, this change has positive dot product with the antigradient of the loss function, see pages 19 and 20 of the Supplemental Material.

As the network is in the balanced state, we expect the symmetric part of $\mathbf{\Omega}$ to be negative-definite, that is for all $\mathbf{v} \neq 0$, $\mathbf{v}^\top \mathbf{\Omega} \mathbf{v} < 0$. As a consequence

$$\begin{aligned} -\langle \Delta \mathbf{r}^\top \rangle \cdot \frac{\partial L}{\partial \mathbf{r}} &= -\frac{1}{\lambda} \langle \Delta \mathbf{r}^\top \rangle \cdot \mathbf{\Omega} \langle \Delta \mathbf{r}^\top \rangle \\ &> 0. \end{aligned}$$

In other words, the change in instantaneous firing rate of the network will have positive dot product with the antigradient of the loss function, thus minimising it.

17. In Section 10 of the Supplementary material (page 26) the argument for $(\mathbf{x} - \hat{\mathbf{x}})$ being proportional to $\mathbf{x}$ could benefit from more substantiation and motivation.

Briefly, the key idea here is that, in the presence of quadratic costs, the readout $\hat{\mathbf{x}}$ will be shorter than the signal $\mathbf{x}$. We have now added the following motivation for this approximation in the supplementary materials:

To see why that is the case, we note that, once the recurrent connections have been learnt, the voltages of the neurons are in the balanced state, i.e., their averages are zero. Taking averages over time for fixed signals, $\mathbf{x}$, we can therefore write,

$$\langle \mathbf{V} \rangle = \langle \mathbf{F}(\mathbf{x} - \hat{\mathbf{x}}) - \mu \mathbf{r} \rangle \approx 0 \tag{2}$$

Multiplying by the decoder from the left and re-arranging, we obtain

$$\begin{aligned} \langle \mathbf{D}\mathbf{F}(\mathbf{x} - \hat{\mathbf{x}}) - \mu \hat{\mathbf{x}} \rangle &\approx 0 &(3)\\ \langle (\mathbf{D}\mathbf{F} + \mu \mathbf{I})(\mathbf{x} - \hat{\mathbf{x}}) \rangle &\approx \mu \langle \mathbf{x} \rangle &(4)\\ \langle (\mathbf{x} - \hat{\mathbf{x}}) \rangle &\approx \mu (\mathbf{D}\mathbf{F} + \mu \mathbf{I})^{-1} \langle \mathbf{x} \rangle &(5) \end{aligned}$$

For sufficiently large μ, the errors will therefore approximately align with $\mathbf{x}$. The costs μ will moreover prohibit $\hat{\mathbf{x}}$ to fully match the size of $\mathbf{x}$, an effect that increases linearly with the size of $\mathbf{x}$.

18. On the following page (section 10, page 27) Why is the fixed point written as $\langle \mathbf{r}\mathbf{x}^\top \rangle$? How is this equal to the expectation of x at the time of a spike?

This is a consequence of the assumption that the input currents are changing slowly, as mentioned on page 27.

> For the sake of mathematical precision, we will here make the assumption that $\mathbf{c}$ is changing slowly, as also stated at the very beginning, section 1.1, and we will proceed with (S.34).

19. In section 12 of the supplementary material, does S.43 imply that the synapses need to learn the mean of the signal?

Indeed, the reviewer is correct, the synapses will need to have a mechanism that lets them keep track of the mean of the signal. More specifically, they should only learn based on deviations from that mean. We have not made any attempt to relate this assumption to the biophysics of synapses. We only emphasize the locality of the learning rules.

(We note, though, that any type of (long-time-scale) adaptation process will act as a high-pass filter, and therefore eliminates the mean, which would be sufficient for the purposes of our learning rules.)

20. In Sec. 13 of the supplementary material: the text explains the idea that the inhibitory population can approximates the activity r of the excitatory population. The idea mentioned in page 33 is that the inhibitory neurons represent the signal encoded by the excitatory population. But the derivation in page 34 proposes that they learn to represent the activity r of the excitatory population, which is not the same quantity – it will be helpful to clarify this. In addition, r varies quite rapidly, on the time scale of the membrane potential following each spike, which may violate the assumption of a slow signal mentioned in section 1.1 of the supplementary material. Does this cause any difficulties?

The key idea here is that, if the inhibitory population can properly track $\mathbf{r}$ along directions in firing rate space that matter for the encoding of the signals, it will automatically also track $\hat{\mathbf{x}}$. The reviewer is correct to note that $\mathbf{r}$ will not just undergo signal-induced changes in directions determined by the decoder $\mathbf{D}$, but also fluctuations in directions orthogonal to the decoder $\mathbf{D}$. However, from the point-of-view of the inhibitory population, these latter fluctuations, which are fast and noisy, are negligible compared to the slower, but much stronger and reliable firing rate changes induced by the signal. The inhibitoryb population therefore learns to track these latter changes, rather than the random fluctuations.

In practice, we have not found any difficulties with this scheme.

Typos and minor comments

21. In page 6, first two rows: the sentence should be corrected (what does 'they' refer to?)
22. In Figures 2 and 4, what does "Error" mean? In Figure 7, is "Error [%]" the same quantity, multiplied by a hundred? It will be helpful to use the same notation in all figures.
23. In page 11 it took me a while to understand what "the length of the decoder weights" means (this terminology is used also in page 10). Perhaps replace by the L2 norm?
24. The first paragraph of the section "Learning in networks with separate excitatory and inhibitory populations" (page 13) refers to equations numbered 1 and 2. These equation numbers seem to be incorrect.
25. In the section on excitatory and inhibitory populations, it will be helpful to mention that both the inhibitory and excitatory neurons follow the dynamics of Eq. (1), with the same time constant.

26. In the section “Learning for correlated inputs” it is desirable to refer early on to the appropriate section of the supplementary text.
27. In page 4 of the supplementary material: “Each filtered spike trains”.
28. In page 9 of the supplementary material (section 4), above condition 3: the statement “A second observation is that the optimal architecture deviates from...” is not clear, because it is detached from the derivation of the recurrent connectivity and is immediately followed by a statement about the feedforward connectivity. A bit more explanation (what is the difference in the architecture and what is the source of this difference) will be helpful.
29. In Section 6.1 of the supplementary material: the sentence on before last at the end of the paragraph is grammatically incorrect (“must be quickly canceled... or otherwise causes”).
30. In the equation at the bottom of page 15 of the supplementary material, the limits of the integrals over tau seem to be incorrect (they should be replaced by minus infinity to t_i).

We thank the reviewer for pointing out all of these typos and minor inaccuracies, which we have now fixed. Concerning remark 22, we note that all errors are plotted as mean-square errors between signal and reconstruction. For all practical purposes, though, these errors are in arbitrary units (as they completely depend on the specific parameters of the simulations) and are only meaningfully compared within a figure, not across figures. We now simply write 'Error [a.u.]' and point out that it is the mean-square error in the legend.
