# Supplementary material for: Learning to represent signals spike by spike
Source: PLoS Comput Biol. 2020 Mar 16;16(3):e1007692. doi: 10.1371/journal.pcbi.1007692 (PMC7135338; doi:10.1371/journal.pcbi.1007692)
Supplement: Attachment — Submitted filename: Referee report.pdf [file pcbi.1007692.s002.pdf]

Referee report: PCOMPBIOL-D-19-01208

The manuscript addresses efficient coding of inputs by spiking neural networks. Theories of efficient coding have a long history in neuroscience. The approach taken here differs from most other works in the assumptions made on the generation of spikes. In the proposed scheme, spikes of individual neurons may appear noisy and stochastic, but in fact every spike is generated by a rule that aims to minimize the reconstruction error of an implicit linear decoder, while taking into account the activity of all others neurons in the network.

The above approach is related to previous works from the same senior authors, in which the goal was to design recurrent networks of spiking neurons that precisely implement certain dynamics. The previous works argued that the emergent solution is one in which internal variables related to the dynamics will be efficiently represent by spikes. In this sense, the present work takes a step backwards, by focusing solely on efficient coding of an external variable. In my opinion this apparent step backwards is in fact very useful, as it allows for a more rigorous treatment of this family of problems.

I appreciated the detailed supplementary text which, despite being highly mathematical in nature, lays out the ideas in a principled and usually clear manner. The exposition of the ideas in the main text, in comparison, is sometimes too vague or sketchy to be comprehensible. There are some aspects of the presentation that can be improved in the supplementary text as well.

My overall assessment is that this work offers an important contribution to the theory of neural coding, and its content is therefore very appropriate for publication in PLoS Computational Biology. However, there are some issues that require additional explanation or correction.

General comments

1. It will be helpful to explain more explicitly how this work differs from previous works by the same authors on optimization of spiking neural networks (Refs 21-24). My understanding is that the main differences are: (a) in the goal (as mentioned above), (b) in the training scheme, i.e. in proposing an online learning rule which is biologically plausible. Even though (b) is stated in the introduction, it is not sufficiently clear from the phrasing that this is the key contribution of the present manuscript.

   In terms of the optimization goal (regardless of the training scheme), it will be helpful to understand whether the goal considered here is a special instance of the problem considered in the previous works. If so, what is the relationship between the solutions for synaptic connectivity obtained in these different works (even if the learning rules are different).

   A specific, somewhat technical difference between the previous works and the present manuscript is that in the former works, the dynamics involved both slow synapses and fast (or instantaneous) synapses, whereas here all synapses are instantaneous - see also comment 4. It will be helpful to comment on this.
2. Likewise, the manuscript proposes two learning algorithms and argues that both are optimal. Do they converge to the same solutions, or at least the same values of the objective functions? If the solutions are not the same, is this because there are multiple local minima? or because of some other reason, such as lack of optimality?

   One of the reasons for a need to address these questions is that the argumentation for convergence to optimal solutions with the voltage-based rules relies on several uncontrolled approximations and assumptions. Hence, numerical testing of its performance relative to other schemes is warranted.

3. The relationship to other works on efficient coding is not sufficiently clear, and is sometimes confusing. Most works on efficient coding focused on the receptive fields of single cells, and not on how the recurrent connectivity can generate jointly an efficient representation by spikes. Accordingly, spiking was mostly considered as generating noise which is independent in different neurons. In this respect, the present work offers an important advancement.

   On the other hand, the present work does not incorporate important lessons that were learned from works that focused on the optimal receptive fields in a population of neurons that encode a multidimensional input:

   a. First, some works on efficient coding considered mutual information rather than reconstruction error as the optimization goal.
   b. Under certain variants of optimization with sparseness constraints, optimal solutions implement ICA on the inputs, not PCA. The solutions obtained here are only sensitive to the covariance matrix of the inputs and therefore do not implement ICA.
   c. Under L2 constraints and without noise in the inputs, networks typically implement whitening without the need for a modified loss function as in Section 12. Here (in the present work) it seems that the whitening is forced into the solution by the choice of the loss function, and this requires some clarification.
   d. Once noise is included in the inputs, it is typically suboptimal to simply whiten the inputs. This was clarified for example by the classical works of Attick and Redlich on encoding of visual stimuli by the retina, as well as others.

   It is possible that the formulation proposed in the present work can be extended to incorporate all these ingredients, but as far as I can see this is not the situation right now. Therefore, it will be helpful to lay out more clearly and correctly the relationship to previous works on encoding of multidimensional inputs and acknowledge the limitations of the present work. The last paragraph of supplementary section 1.4 is very confusing in this respect.

4. The learning of feedforward connections is chosen to be slow compared to the learning of recurrent connections, and this is apparent already in Figure 2 but not discussed (unless I missed this). Later on, in at least two instances, the difference in time scales is mentioned as an important requirement or consequence, and time scale separation is used in some of the argumentation in the supplementary material. However, as far as I understand, the results don't demonstrate that successful learning occurs only if the time scale of feedforward learning is slow compared to the recurrent learning, and it will be helpful to comment directly on this question.

More specific comments

5. In the network dynamics (Eq. 1) synaptic currents are instantaneous. This should be stated explicitly. The phrasing in page 16 misrepresents the situation, since the postsynaptic current generated by an incoming spike is a delta function, not a jump followed by exponential decay. The *voltage* jumps and decays exponentially following a spike, not the *current.* This dynamics of the voltage has nothing to do with synaptic dynamics.

6. I recommend to briefly mention the basic requirements from an optimal decoder in the main text, before discussing the current and voltage based learning rules. These requirements are currently derived in sections 2-5 of the supplementary material and summarized in section 6. It will be very helpful to clearly lay out the requirements in the main text, preferably using some equations. Otherwise, it remains unclear while reading the main text alone that optimization of the error entails certain concrete requirements on the recurrent and feedforward connectivity and (implicitly) on the decoder. Consequently, it is not clear to the reader in what sense the local learning rules derived later achieve optimal performance.

7. The discussion on the current based decoder lacks concreteness in the main text. It will be helpful to provide some equations involving g, in similarity to Eqs. (5) and (6) that are provided for the voltage based rule. Otherwise it is not clear that the idea of balancing the currents translates into a specific synaptic learning rule. It will also be helpful to refer to the appropriate supplementary sections earlier in the text.
8. Likewise, it will be helpful to mention what are the relevant supplementary sections early on when introducing the voltage based learning, to clarify that there is a systematic derivation behind the proposed rules, which are only qualitatively motivated in the main text.
9. The argumentation at the top of page 9 is difficult to follow and seems circular. The problem starts with "Since the connectivity structure dictates that the voltage becomes a function of the global coding error": isn't this part of what needs to be shown? How does this follow from the stationary state arising from rule (5)?
10. If I am not mistaken, the exposition in the main manuscript fails to acknowledge the need to show that the rules converge to a decoder that minimizes the loss function (Sections 8.3 and 10.2). This is something that I was puzzled about while reading the main text, and became clear only while reading carefully through the supplementary text. I recommend to mention this explicitly.
11. Fig. 4 caption: the text on panel A discusses an STDP rule depending on the timing of pre- and post- synaptic spikes. However the recurrent learning rule discussed in the text involves the presynaptic spike and the postsynaptic voltage, while the feedforward rule involves a postsynaptic spike and the presynaptic continuous input. Please clarify.
12. The results on more realistic synaptic dynamics (page 16) are not sufficiently clear and detailed.

    a. Please explain clearly that the synapses were instantaneous up to that point (see also comment 1).
    b. The phrasing "transmission delays" is confusing, as it suggests a simple delay of the synaptic current without any other modification in its structure. A better phrasing will be something along the lines of "realistic, non-instantaneous synaptic currents" with reference to panel D of Figure 7.
    c. In panel C, the ratio between the error with 2ms delay and 0 ms delay is very large, more than an order of magnitude. In what sense then is the degradation in performance small, as stated in page 16? And how does the error compare to its value at the beginning of training? Does it still improve with increase of the network size?.

13. The Pseudo-Code in the supplementary text may be helpful for reproducing the results. It is less useful if one wishes to simply understand in mathematical terms what were the dynamics of neurons and synapses in each simulation. It's difficult to do so now because equations are scattered in the main manuscript and in the multiple sections of the supplementary material. Therefore, I suggest to add a summary of the equations used, either in the supplementary text or as a methods section. Either repeat the equations or refer to them by number, but specify clearly what were the equations that were used in each Figure.
14. I didn't fully understand the logic of Sec. 5.2. I understand that greedy optimization of the spike timing yields Eqs. S.10 and S.11. But if the threshold is instead fixed at some value that deviates from optimality, is it obvious that the best choice for the decoder weights is the one that obeys these two equations?
15. In page 19 (section 8.3), at the left hand side of the equations, why is there no average symbol on the voltages? In addition, the argumentation is confusing since $<V>$ is assumed to be close to zero, but then naively $<\Delta V>$ will remain close to zero as well. I suppose that all terms in

the equation are small but in the last line of the equation <*V*> can somehow be shown to be small compared to the first term. This is not done in a sufficiently convincing manner.

16. In the last paragraph of page (19) of the supplementary material I did not understand the argument. If r (not <*V*>) would change in the direction of the anti gradient with respect to r, the argument would be clear.
17. In Section 10 of the Supplementary material (page 26) the argument for $(x - \hat{x})$ being proportional to x could benefit from more substantiation and motivation.
18. On the following page (section 10, page 27) Why is the fixed point written as $\langle r\, x^T \rangle$? How is this equal to the expectation of x at the time of a spike?
19. In section 12 of the supplementary material, does S.43 imply that the synapses need to learn the mean of the signal?
20. In Sec. 13 of the supplementary material: the text explains the idea that the inhibitory population can approximates the activity r of the excitatory population. The idea mentioned in page 33 is that the inhibitory neurons represent the signal encoded by the excitatory population. But the derivation in page 34 proposes that they learn to represent the activity r of the excitatory population, which is not the same quantity – it will be helpful to clarify this. In addition, r varies quite rapidly, on the time scale of the membrane potential following each spike, which may violate the assumption of a slow signal mentioned in section 1.1 of the supplementary material. Does this cause any difficulties?

Typos and minor comments

21. In page 6, first two rows: the sentence should be corrected (what does 'they' refer to?)
22. In Figures 2 and 4, what does "Error" mean? In Figure 7, is "Error [%]" the same quantity, multiplied by a hundred? It will be helpful to use the same notation in all figures.
23. In page 11 it took me a while to understand what "the length of the decoder weights" means (this terminology is used also in page 10). Perhaps replace by the L2 norm?
24. The first paragraph of the section "Learning in networks with separate excitatory and inhibitory populations" (page 13) refers to equations numbered 1 and 2. These equation numbers seem to be incorrect.
25. In the section on excitatory and inhibitory populations, it will be helpful to mention that both the inhibitory and excitatory neurons follow the dynamics of Eq. (1), with the same time constant.
26. In the section "Learning for correlated inputs" it is desirable to refer early on to the appropriate section of the supplementary text.
27. In page 4 of the supplementary material: "Each filtered spike trains".
28. In page 9 of the supplementary material (section 4), above condition 3: the statement "A second observation is that the optimal architecture deviates from…" is not clear, because it is detached from the derivation of the recurrent connectivity and is immediately followed by a statement about the feedforward connectivity. A bit more explanation (what is the difference in the architecture and what is the source of this difference) will be helpful.
29. In Section 6.1 of the supplementary material: the sentence on before last at the end of the paragraph is grammatically incorrect ("must be quickly canceled… or otherwise causes").
30. In the equation at the bottom of page 15 of the supplementary material, the limits of the integrals over tau seem to be incorrect (they should be replaced by minus infinity to t_i).
